# Simultaneous Representation Learning of Multi‐Omics and Clinical Outcome Data via a Supervised Knowledge‐Guided Bayesian Factor Model

**DOI:** 10.1002/sim.70570

**Published:** 2026-04-26

**Authors:** Qiyiwen Zhang, Changgee Chang, Chong Jin, Li Shen, Qi Long

**Affiliations:** ^1^ Department of Medicine University of Pittsburgh Pittsburgh Pennsylvania USA; ^2^ Department of Biostatistics and Health Data Science Indiana University Bloomington Bloomington Indiana USA; ^3^ Mathematical Sciences New Jersey Institute of Technology Newark New Jersey USA; ^4^ Department of Biostatistics, Epidemiology and Informatics University of Pennsylvania Philadelphia Pennsylvania USA

**Keywords:** Bayesian model, factor analysis, knowledge‐guided, multi‐omics AD, simultaneous representation learning

## Abstract

With the advent of high‐throughput techniques, multi‐omics data and various clinical outcomes have been collected for a range of diseases. Multi‐omics data play a crucial role in uncovering complex biological processes, yet simultaneous representation learning of such high‐dimensional, heterogeneous multi‐modality data along with clinical outcomes remains limited. To address this gap, we propose a supervised knowledge‐guided Bayesian factor model for integrative analysis of multi‐omics and clinical outcome data. The proposed method simultaneously extracts an informative low‐dimensional representation and predicts one or more clinical outcomes of interest. The two‐level adaptive shrinkage in the novel hierarchical priors allows for the identification of both active modalities and features, resulting in a biologically meaningful structural identification of the high‐dimensional data. Moreover, the method is robust to noisy edges in biological graphs that do not align with ground truth. Finally, the proposed method can handle different data types including both continuous and categorical data. Extensive simulation studies and real data analyses of Alzheimer's disease (AD) data demonstrate the advantages of the proposed approach over existing methods. Notably, our analysis of multi‐omics and imaging phenotype data from ADNI provides meaningful insights into the underlying biological mechanisms of AD.

## 1 Introduction

The advancement of high‐throughput sequencing technologies has enabled comprehensive profiling across genomics, transcriptomics, proteomics, and other omics data. Nowadays, multi‐omics data play a critical role in precision medicine [1], with numerous studies demonstrating its ability to enhance our understanding of disease etiology and progression across diverse areas, including genomic region annotation [2], regulatory network analysis [3], risk gene or biomarker identification [4], and prognosis prediction [5].

However, the integrative analysis of multi‐omics data still presents several significant analytical challenges. For instance, consider the Alzheimer's Disease (AD) dataset, which we revisit later, consisting of 414 patients. This dataset includes single nucleotide polymorphism (SNP) data, gene expression data, metabolomics data, and clinical outcomes such as Mini‐Mental State Exam (MMSE) scores. A key concern is the relatively small number of patients compared to the large number of features, necessitating dimensionality reduction. Additionally, the structured nature of multi‐omics data suggests that modalities may contain both shared and distinct information relevant to biological processes, emphasizing the importance of considering modality‐level sparsity alongside feature‐level sparsity. Furthermore, AD is recognized as a multifactorial disease, influenced by harmful mutations and alterations not only across various omic levels but also at the pathway level, where genes or gene products functionally interact. Incorporating such knowledge, often described by graphs, can help identify relevant features and enable biologically meaningful dimension reduction. However, limited research exists on modeling multi‐omics data in this way. In practice, dimension reduction is not the end of the analysis; it is often followed by prediction tasks such as regression. In such cases, the associations between AD outcomes and original modalities should not be neglected, as this additional association information can benefit in terms of both dimension reduction and outcome prediction. Yet, little attention has been given to developing a principled modeling framework that performs joint learning tasks. Therefore, novel simultaneous learning methods are needed to address these analytical challenges.

Factor analysis, a powerful technique for reducing high‐dimensional data to a lower‐dimensional space, has found extensive use in the integrative analysis of multi‐omics data [6]. A number of methods have emerged in the field of factor analysis. Ref. [7] introduced Joint and Individual Variation Explained (JIVE), employing a sequential approach to first identify globally shared latent factors and then select modality‐specific latent factors, conditioned on the shared factors. Other similar methods, such as those proposed by [8, 9], also focus on globally shared and modality‐specific latent factors. Ref. [10] developed Structural Learning and Integrative Decomposition (SLIDE), a significant contribution that characterizes not only globally shared and modality‐specific latent factors but also partially shared latent factors. In contrast to these frequentist methods, multiple Bayesian factor analysis approaches have been developed [11, 12, 13, 14, 15, 16, 17]. Among these methods, [17] proposed a pathway‐guided Bayesian factor model for the simultaneous identification of subgroups and key molecular features. Another example is Multi‐Omics Factor Analysis (MOFA), proposed by [16]. MOFA is tailored to multi‐omics data, accommodating various latent structures, including globally shared, partially shared, and modality‐specific factors, while handling diverse data types effectively. None of the methods mentioned thus far has the capability to integrate information from outcome variables, thus hindering the simultaneous learning of factor analysis and predictive models such as regression. In recent years, multiple methodological frameworks have been developed for the integrative analysis of multi‐omics data and associated clinical outcomes [18, 19, 20]. In particular, [18] extended their prior work, JIVE, to introduce supervised Joint and Individual Variation Explained (sJIVE). This innovative approach enables the simultaneous learning of factor and regression models, offering promise in addressing this limitation. Nonetheless, the formulation of sJIVE may pose challenges in effectively handling multiple outcome variables. Importantly, all aforementioned methods cannot incorporate biological graph knowledge into the analysis.

Graph knowledge has demonstrated utility in linear regression and variable selection [21, 22]. Ref. [23] explored various forms of biological knowledge and databases, including the Kyoto Encyclopedia of Genes and Genomes (KEGG) [24] and Mummichog [25]. Recently, the application of graph knowledge has been extended to factor analysis. For instance, [15] incorporated graph knowledge using Markov random field (MRF) priors. However, a key problem with MRF priors is the phase transition problem [26], which can lead to extreme variable selection dependency, either too weak or too strong, across most of the tuning parameter space, thereby increasing the computational burden of the tuning process. Refs. [27, 28] employed graph Laplacian priors, but both approaches impose a diagonal dominance constraint when incorporating the graph, limiting the information offered by the biological graph knowledge. Furthermore, while [28] developed a framework to account for noisy graphs, this extension hinders the model's capacity to handle discrete data types, such as binomial data. Consequently, there remains a need to develop statistical methods with more effective graph‐incorporated priors.

Motivated by the limitations of existing methods and the analysis of multi‐omics and imaging phenotype data in ADNI, we have developed a supervised graph‐guided Bayesian factor analysis model (sGBFA) capable of handling multi‐omic data across different data types. Our work offers several key contributions. First, the proposed model induces sparsity at both the feature level and the modality level. This dual‐shrinkage approach allows for efficient variable selection within individual modalities while simultaneously enabling structural learning across multiple modalities. Additionally, modality‐level sparsity categorizes factors into three distinct types, enhancing the interpretability of the results. Specifically, these factors include those shared across all modalities, those shared by a subset of modalities, and those unique to a single modality. Second, the model's simultaneous representation learning framework allows for the flexible integration of outcome variables, such as cognitive scores and imaging phenotypes. This additional information enhances the identification of low‐dimensional representations, which, in turn, improves regression predictions. Third, sGBFA incorporates the latest Wishart prior developed in our recent work [29], designed to mitigate the MRF phase transition problem and overcome the diagonal constraint issue. This prior also provides the flexibility to smoothly control variable selection dependency, thereby increasing robustness to noisy edges. Finally, the combination of double shrinkage and graph knowledge enhances the biological interpretability of the resulting low‐dimensional representations.

The remainder of the article is organized as follows. Section 2 describes the proposed method. Section 3 explores various simulation scenarios to evaluate the performance and robustness of our method. Section 4 applies sGBFA to analyze ADNI data, followed by discussions in Section 5.

## 2 Methodology

### 2.1 Supervised Graph‐Guided Factor Model

Suppose we have n samples with the multi‐modal data matrix $X={\left[{\left(X^{\left(1\right)}\right)}^{T},\ldots ,{\left(X^{\left(H\right)}\right)}^{T}\right]}^{T}$ where $X^{\left(h\right)}$ is a $p_{h}\times n$ data matrix for the h‐th modality with $p_{h}$ features. The distributional parameters $\mu^{\left(h\right)}\in {\mathbb{R}}^{p_{h}\times n}$, which are related to the expectation of $X^{\left(h\right)}$ through data‐specific link functions, are assume to have the following low‐dimensional representation:

$$\mu^{\left(h\right)}=m^{\left(h\right)}1^{T}+W^{\left(h\right)}Z,\;h=1,\ldots ,H,$$

where $m^{\left(h\right)}\in {\mathbb{R}}^{p_{h}\times 1}$ is the location parameter, $W^{\left(h\right)}\in {\mathbb{R}}^{p_{h}\times L}$ is the factor loading matrix, $Z\in {\mathbb{R}}^{L\times n}$ is the latent factor matrix and 1 stands for a vector of ones. L is the number of latent factors. For example, for a Gaussian variable we have $\mu_{\mathrm{ji}}^{\left(h\right)}=E\left[x_{\mathrm{ji}}^{\left(h\right)}\right]$; for a binomial variable we have $\mu_{\mathrm{ji}}^{\left(h\right)}=\text{logit}\left(E\left[x_{\mathrm{ji}}^{\left(h\right)}\right]\right)$. Concatenating all modalities by row for $\mu^{\left(h\right)}$, $W^{\left(h\right)}$ and $m^{\left(h\right)}$ gives a unified representation $\mu =m1^{T}+\mathrm{WZ}$. Finally, we use $(x_{\mathrm{ji}}^{\left(h\right)}$, $w_{\mathrm{jl}}^{\left(h\right)},z_{\mathrm{li}})$ to denote scalar entries of the corresponding matrices, and $x_{j}^{\left(h\right)}$ to denote the j‐th row of $X^{\left(h\right)}$ throughout the article.

**FIGURE 1:**
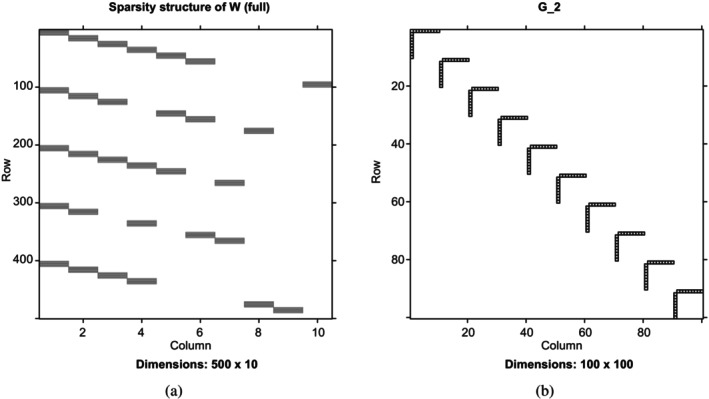
(a) Illustrates one potential sparsity structure of the factor loading matrix (p=500 features, L=10 latent factors, and H=5 modalities). (b) Visualizes a corresponding graph structure for a single modality (p=100 features).

One appealing property of the proposed model is its ability to incorporate the biological graph information, denoted as 𝒢=⟨P,E⟩, with a set of nodes (features) P and a set of edges E, which characterize interactions among these features. It is important to note that we focus solely on intra‐modality graphs; inter‐modality graphs are not considered in this context. A symmetric matrix D is said to be compatible with 𝒢, provided that the off‐diagonal entry $d_{\mathrm{ij}}\neq 0$ if and only if there is an edge between the i‐th node and j‐th node in the graph 𝒢. Figure 1b presents the adjacency matrix of the graph used for a single modality with 100 features. Additional graph visualizations are provided in Supporting Information: Section 3.

The incorporation of graph knowledge encourages to select a group of features that work collaboratively. For example, the existence of an edge between the j‐th and k‐th features within modality h implies $\mathrm{Cov}\left(x_{j}^{\left(h\right)},x_{k}^{\left(h\right)}\right)\neq 0$. Given the standard assumption $\mathrm{Cov}\left(x_{j}^{\left(h\right)},x_{k}^{\left(h\right)}\right)=\sum_{l}w_{\mathrm{jl}}^{\left(h\right)}w_{\mathrm{kl}}^{\left(h\right)}$ in the factor analysis setting, it indicates there exists at least one $l_{0}$ for which $w_{jl_{0}}^{\left(h\right)}w_{kl_{0}}^{\left(h\right)}\neq 0$. Hence, the presence of an edge between the j‐th and k‐th features naturally leads to the joint selection of the pair $\left(w_{jl_{0}}^{\left(h\right)},w_{kl_{0}}^{\left(h\right)}\right)$.

Secondly, the proposed model facilitates dual‐shrinkage at both the feature and modality levels, enabling certain columns in the modality‐specific $W^{\left(h\right)}$ matrices to be exactly zero. This combination of block‐sparsity and individual‐sparsity enhances the interpretability of the resulting model. To better illustrate the group structure of factor loadings, we define three types of latent factors based on the block‐sparsity of W. Let 0 be a zero vector and $w_{l}^{\left(h\right)}$ refer to the l‐th column of $W^{\left(h\right)}$. Factors are shared by all modalities if $w_{l}^{\left(h\right)}\neq 0$ for ∀h; factors are shared by a subset (more than one) of modalities if $w_{l}^{\left(h\right)}\neq 0$ for at least two h∈{1,…,H}, and factors are specific to a modality if $w_{l}^{\left(h\right)}\neq 0$ for a single h∈{1,…,H}. From a biological perspective, factors shared across all or a subset of modalities capture the joint associations among these modalities, while modality‐specific factors represent systematic variations unique to a particular modality, independent of the others. For instance, previous studies have demonstrated strong associations between SNP data and gene expression data for certain genes [30], supporting the plausibility of modeling shared patterns across these two modalities. Figure 1a illustrates a potential group structure of the factor loading matrix W in the setting of L=10 latent factors and H=5 modalities, each containing 100 features. Nonzero entries are shown in black, while zero entries appear in white. Each black block corresponds to five features. Based on the definitions above, the first two columns represent latent factors shared by all five modalities. Columns 3−8 correspond to latent factors shared by subsets of modalities. For instance, Columns 3 and 4 are shared by four modalities, Columns 5 and 6 by three modalities, and Columns 7 and 8 by two modalities. The final two columns represent modality‐specific latent factors.

In addition, the proposed framework allows for a joint learning of the factor model. Suppose $Y\in {\mathbb{R}}^{p_{y}\times n}$, with each row corresponding to an outcome vector $y_{j}=\left(y_{j1},\ldots ,y_{\mathrm{jn}}\right)$. The regression models that take the latent factors as predictors are given by:

$$y_{j}=\mu_{y}+{ℰ}_{j}=\beta_{j0}+Z^{T}\beta_{j}+{ℰ}_{j},j=1,\ldots ,p_{y}$$

where $\beta_{j0}$, $y_{j}\in {\mathbb{R}}^{n\times 1}$, $\beta_{j}=\left(\beta_{j1},\ldots ,\beta_{\mathrm{jL}}\right)\in {\mathbb{R}}^{L\times 1}$ and ${ℰ}_{j}\in {\mathbb{R}}^{n\times 1}$ refer to the intercept, response, regression coefficients and Gaussian errors (following $𝒩\left(0,\sigma_{y_{j}}^{2}\right)$). Traditionally, learning latent factors and building a regression model with the estimated latent factors are two sequential steps. However, this approach overlooks the inherent association between the outcome and modalities. By contrast, our proposed approach involves the simultaneous learning of factor and regression models during the training phase, capitalizing on the intrinsic connections between modalities and outcomes. This integrated learning strategy enables us to obtain more robust estimations for W, Z, and $\beta_{j}$ collectively. Consequently, it results in more informative low‐dimensional representations of the original data and promotes the accuracy of predictions.

Finally, the proposed model is able to handle different data types including Gaussian, binomial, and negative binomial. We assume conditional independence for the likelihood function:

$$\pi \left(X|\mu \right)=\prod_{h}\;\prod_{j}\;\prod_{i}\pi^{\left(h\right)}\left(x_{\mathrm{ji}}^{\left(h\right)}|\mu_{\mathrm{ji}}^{\left(h\right)}\right),$$

where $\pi^{\left(h\right)}\left(\cdot \right)$ refers to the density function for the modality h. For Gaussian data, we have

$$\pi^{\left(h\right)}\left(x_{\mathrm{ji}}^{\left(h\right)}|\mu_{\mathrm{ji}}^{\left(h\right)},\rho_{j}^{\left(h\right)}\right)=\frac{{\left(\rho_{j}^{\left(h\right)}\right)}^{1/2}}{\sqrt{2\pi }}e^{-\rho_{j}^{\left(h\right)}{\left(x_{\mathrm{ji}}^{\left(h\right)}-\mu_{\mathrm{ji}}^{\left(h\right)}\right)}^{2}/2},$$

where $\rho_{j}^{\left(h\right)}$ refers to the precision with $\rho_{\mathrm{ji}}^{\left(h\right)}\equiv \rho_{j}^{\left(h\right)}$ for 1≤i≤n. For binomial data, we have

π(h)xji(h)|μji(h),nj(h)=nj(h)xji(h)eμji(h)xji(h)1+eμji(h)nj(h),

for $x_{\mathrm{ji}}^{\left(h\right)}\in \left\{0,1,\ldots ,n_{j}^{\left(h\right)}\right\}$, where $n_{j}^{\left(h\right)}$ refers the number of trials with $n_{j}^{\left(h\right)}\equiv n_{\mathrm{ji}}^{\left(h\right)}$. For negative binomial data,

π(h)xji(h)|μji(h),rj(h)=rj(h)+xji(h)−1xji(h)eμji(h)xji(h)1+eμji(h)rj(h)+xji(h),

for $x_{\mathrm{ji}}^{\left(h\right)}\in \left\{0,1,\ldots \right\}$, where $r_{j}^{\left(h\right)}$ refers to the failure parameter with $r_{\mathrm{ji}}^{\left(h\right)}\equiv r_{j}^{\left(h\right)}$. The probability $p_{\mathrm{ji}}^{\left(h\right)}$ of binomial density or negative binomial density is connected with $\mu_{\mathrm{ji}}^{\left(h\right)}$ through the logit function.

### 2.2 Prior Specifications

For the sake of simplicity, we use a generic notation C to denote the normalizing constant that may vary across formulas. The superscript y refers to the parameters associated with the outcome modality. Both factor loadings W and regression coefficients $\beta_{\mathrm{jl}}$ are independently distributed with Laplace priors:

(1) $$\begin{array}{cc} \log\pi \left(w_{\mathrm{jl}}^{\left(h\right)}|\lambda_{\mathrm{jl}}^{\left(h\right)},\phi_{l}^{\left(h\right)}\right) & =C+\log\left(\lambda_{\mathrm{jl}}^{\left(h\right)}\phi_{l}^{\left(h\right)}\right)-\phi_{l}^{\left(h\right)}\lambda_{\mathrm{jl}}^{\left(h\right)}\left|w_{\mathrm{jl}}^{\left(h\right)}\right|,\\ \log\pi \left(\beta_{\mathrm{jl}}|\lambda_{\mathrm{jl}}^{y}\right) & =C+\log\lambda_{\mathrm{jl}}^{y}-\lambda_{\mathrm{jl}}^{y}\left|\beta_{\mathrm{jl}}\right|. \end{array}$$

The primary distinction between the two aforementioned priors lies in the group shrinkage parameter $\phi_{l}^{\left(h\right)}$, which governs modality‐level sparsity, while $\lambda_{\mathrm{jl}}^{\left(\cdot \right)}$ represents individual shrinkage parameters. This prior formulation, particularly with the introduction of $\phi_{l}^{\left(h\right)}$, facilitates learning the group structure of multi‐modal data by estimating different types of latent factors discussed in 2.1. In contrast to existing methods such as [18], which assume that latent factors are either globally shared or specific to individual modality, the inclusion of $\phi_{l}^{\left(h\right)}$ naturally extends this assumption to consider latent factors that are partially shared across a subset of modalities. This extension allows for the identification of a more intricate yet realistic data structure. For convenience, we use the notation $\Phi \in {\mathbb{R}}^{H\times L}$, $\Lambda^{\left(h\right)}\in {\mathbb{R}}^{p_{h}\times L}$, $\Lambda^{y}\in {\mathbb{R}}^{p_{y}\times L}$ to denote the group shrinkage parameters, the individual shrinkage parameters for $W^{\left(h\right)}$, and the individual shrinkage parameters for the regression coefficients, respectively.

For the group shrinkage parameter $\phi_{l}^{\left(h\right)}$, we assign the gamma prior:

(2) $$\log\pi \left(\phi_{l}^{\left(h\right)}\right)=C+\left(a_{\phi }-1\right)\log\phi_{l}^{\left(h\right)}-b_{\phi }\phi_{l}^{\left(h\right)},$$

independently across l=1,…,L and h=1,…,H. Shape parameter $a_{\phi }$ and rate parameter $b_{\phi }$ are pre‐specified. This gamma distribution still yields to the conjugate prior even after using data augmentation techniques for $w_{\mathrm{jl}}^{\left(h\right)}$.

Let $\Lambda^{\mathrm{mod}}$ be the concatenation (by row) of $\Lambda^{\left(h\right)}$ for 1≤h≤H. The priors on the individual shrinkage parameters are separated for ${𝒜}^{\mathrm{mod}}=\log\Lambda^{\mathrm{mod}}$ (Gaussian distribution), related to factor loadings, and $\Lambda^{y}$ (gamma distribution), related to regression coefficients:

(3) $$\begin{array}{r} \begin{array}{l} \log\pi \left({𝒜}^{\mathrm{mod}}|\Omega \right)=C+\frac{L}{2}\log\left|\Omega \right|-\frac{1}{2\nu_{2}}\sum_{l}{\left({\tilde{\alpha }}_{l}^{\mathrm{mod}}\right)}^{T}\Omega {\tilde{\alpha }}_{l}^{\mathrm{mod}},\\ \log\pi \left(\Lambda^{y}\right)=C+\left(a_{\lambda }-1\right)\sum_{j,l}\log\lambda_{\mathrm{jl}}^{y}-b_{\lambda }\sum_{j,l}\lambda_{\mathrm{jl}}^{y}, \end{array} \end{array}$$

where ${\tilde{\alpha }}_{l}^{\mathrm{mod}}=\alpha_{l}^{\mathrm{mod}}-\nu_{1}1$ with $\alpha_{l}^{\mathrm{mod}}$ referring to the l‐th column of the log transformed shrinkage parameter matrix ${𝒜}^{\mathrm{mod}}$, Ω is the precision matrix (up to a constant), through which the graph knowledge is incorporated. $\nu_{1}$, $\nu_{2}$, $a_{\lambda }$ and $b_{\lambda }$ are user‐defined parameters, which could be either pre‐specified or tuned. Note that the prior mean $\nu_{1}$ would specify the overall shrinkage level of the factor loadings and the prior variance $\nu_{2}$ controls the level of confidence on such prior. When $\nu_{2}\to 0$, individual shrinkage parameters of the factor loadings are going to concentrate around $\nu_{1}$, less adaptive to the values of W.

Ω plays a key role in incorporating the underlying graph knowledge. Specifically, a constrained Wishart prior assigned to Ω has the following form:

(4) $$\begin{array}{rr} \log\pi \left(\Omega \right) & =C+\frac{\eta \left(1+\epsilon \right)}{2}\log\left|\Omega \right|\\ & -\;\frac{\eta }{2}\mathrm{tr}\left(\left(11^{T}+\mathrm{\epsilon I}\right)\Omega \right)-\infty 1\left(\Omega \notin {ℳ}_{𝒢}\right), \end{array}$$

where η and ϵ are positive, ${ℳ}_{𝒢}$ is a set of positive definite symmetric matrices compatible with the graph 𝒢. We found that this prior possesses better properties than its competitors in terms of following perspectives. First, our prior is able to control the level of correlation of the shrinkage parameters explicitly by ϵ which alleviates the problem of the phase transition phenomenon possessed by the MRF priors [15, 31]. Although according to [31], one solution to the phase transition problem is to restrict the smoothness tuning parameter to stay below a certain threshold, but this limits the information offered by the graph. If the smoothness tuning parameter is set above the threshold, it can lead to excessive dependence and decrease the robustness to noisy edges that incorrectly describe the ground‐truth structures of parameters. For sGBFA, the off‐diagonal entries of $\Omega^{-1}$ are encouraged to concentrate around $\frac{1}{1+\epsilon }$ as the mode of the unconstrained (to the underlying graph) prior for Ω is ${\left(\frac{11^{T}+\mathrm{\epsilon I}}{1+\epsilon }\right)}^{-1}$. By adjusting ϵ, one can directly control the level of dependence. Second, η allows us to control over the level of beliefs in the prior 4. The larger η indicates the posterior of Ω is more dependent on the prior and less impacted by the data. Finally, unlike priors in [27, 28] requiring Ω to be diagonal dominant, our prior doesn't have any constraint on the diagonal entries, which results in a more flexible matrix space compatible with the underlying graph 𝒢.

To complete prior specifications, we use the Gamma prior for precision parameter $\rho_{y_{j}}=1/\sigma_{y_{j}}^{2}$ of the outcome $y_{j}$ and Gaussian prior for the location vector m, the intercept vector $\beta_{0}={\left\{\beta_{j0}\right\}}_{1\leq j\leq p_{y}}$ of the outcome latent factor Z, which is given by:

$$\begin{array}{rr} & \log\pi \left(m\right)=C-\frac{1}{2\sigma_{m}^{2}}m^{T}m,\\ & \log\pi \left(Z\right)=C-\frac{1}{2}\sum_{l,i}z_{\mathrm{li}}^{2},\\ & \log\pi \left(\beta_{0}\right)=C-\frac{1}{2\sigma_{b}^{2}}\beta_{0}^{T}\beta_{0},\\ & \rho_{y_{j}}∼𝒢\left(\frac{\zeta_{j}^{y}}{2},\frac{\zeta_{j}^{y}}{2}\right), \end{array}$$

where $\sigma_{m}^{2}$ and $\sigma_{b}^{2}$ are the prior variance for each coordinate of m and $\beta_{0}$, respectively; $\zeta_{j}^{y}$ s a user‐specified parameter that controls the shape and rate of the Gamma distribution.

### 2.3 MCMC Algorithm and Tuning Procedure

We develop an efficient MCMC algorithm to explore the following full posterior distribution of parameters:

(5) $$\begin{array}{cc} & \pi \left(W,B,Z,m,\beta_{0},\rho ,\rho_{y},\Lambda^{y},{𝒜}^{\mathrm{mod}},\Phi ,\Omega |X,Y\right)\\ & \propto \pi \left(X|m,W,Z,\rho \right)\pi \left(Y|\beta_{0},B,Z,\sigma_{y}\right)\pi \left(m\right)\pi \left(\beta_{0}\right)\pi \left(Z\right)\pi \left(\rho \right)\pi \left(\rho_{y}\right)\\ & \times \pi \left(W|{𝒜}^{\mathrm{mod}},\Phi \right)\pi \left(\Phi \right)\pi \left({𝒜}^{\mathrm{mod}}|\Omega \right)\pi \left(\Omega \right)\pi \left(B|\Lambda^{y}\right)\pi \left(\Lambda^{y}\right), \end{array}$$

where $B\in {\mathbb{R}}^{p_{y}\times L}$ refers to the regression coefficient matrix with each row corresponding to $\beta_{j}$, $\rho =\left\{\rho_{j}^{\left(h\right)}\right\}$ for 1≤h≤H and $1\leq j\leq p_{h}$ is the precision vector of multi‐omics modalities and $\rho_{y}={\left\{\rho_{y_{j}}\right\}}_{1\leq j\leq p_{y}}$ is the precision vector of outcomes. While most parameters can be sampled with the Gibbs sampler, the shrinkage parameters ${𝒜}^{\mathrm{mod}}$ need to be sampled via a Metropolis‐Hastings (MH) algorithm. The details of the computation derivations can be found in Supporting Information: Section 1.

We use a variant of deviance information criteria (DIC) [32] to select the tuning parameters. Let $D=\left[X^{T},Y^{T}\right]$ be the full matrix containing both the multi‐omics modalities and the outcome modality, and let $\hat{U}=\left[{\hat{\mu }}^{T},{\hat{\mu }}_{y}^{T}\right]$ denote the concatenated estimates of the underlying mean structure, where

$$\begin{array}{rr} \hat{\mu } & =\frac{1}{T}\sum_{t}\mu_{t}\;\text{with}\;\mu_{t}=m_{t}+W_{t}Z_{t},\\ {\hat{\mu }}_{y} & =\frac{1}{T}\sum_{t}{\left(\mu_{y}\right)}_{t}\;\text{with}\;{\left(\mu_{y}\right)}_{t}={\left(\beta_{0}\right)}_{t}+B_{t}Z_{t}. \end{array}$$

The DIC is given by:

(6) $$\mathrm{DIC}\left(\nu_{1},\nu_{2},L\right)=-2l\left(D,\hat{U}\right)+4\times \text{diff},$$

where $\text{diff}=l\left(D,\hat{U}\right)-E\left[l\left(D,U\right)\right]$ with the log‐likelihood l(·,·) and the mean of log‐likelihood over the MCMC samples $E\left[l\left(D,U\right)\right]=\frac{1}{T}\sum_{t}l\left(D,U_{t}\right)$. Here, $W_{t}$, $Z_{t}$, $m_{t}$, ${\left(\beta_{0}\right)}_{t}$ and $B_{t}$ refer to the t‐th MCMC samples. A grid search is used to identify the optimal combination of $\left(\nu_{1},\nu_{2},L\right)$ that yields the smallest DIC. We fix η=10 and ϵ=0.2 in our simulation studies where η reflects the prior confidence of the biological graph knowledge and ϵ controls the level of correlation of the individual shrinkage parameters.

## 3 Simulation

We conducted extensive simulation studies to evaluate the performance of the proposed method. For comparison, we included several state‐of‐the‐art approaches: SBFA [27], SLIDE [10], MOFA [16], JIVE [7], and sJIVE [18]. Among these methods, sJIVE is the only one capable of incorporating outcome information, whereas SBFA is the only method that integrates graph knowledge. Two simulation settings were designed to highlight different aspects of our framework. Simulation I, which excludes outcome variables, aims to assess the proposed method's effectiveness in dimension reduction under a variety of conditions. This setting evaluates the benefits of incorporating prior graph knowledge, the ability to accommodate heterogeneous group structures, and the robustness of the model to misspecified graph knowledge. Simulation II extends the evaluation to scenarios with outcome variables, focusing on both predictive performance and dimension reduction of the proposed model in such integrative framework. All simulation results are summarized over 50 repeated samplings. The implementation of sGBFA is publicly available at https://github.com/Qiyiwenzhang/sGBFA. Additional simulation studies exploring alternative designs, including different (p,n) combinations, varying noise levels, and a comparative case study between sGBFA and RABFA [29], are presented in Supporting Information: Section 2.

In Simulation I, we consider H=5 modalities with n=200 samples and p=500 features (100 features per modality). The simulation scenarios vary with respect to the group structure of factor loading matrix W and the data type. Four data types are examined: Gaussian, binary, binomial and mixed. In the mixed data setting, two modalities are Gaussian, two are binomial, and one is binary. Regarding the group‐structure of factor loadings, we define four configurations of W, based on the composition of latent factors: **full**, **ai**, **pi**, and **ap**. In the **full case**, the number of factors is L=10, including two factors shared by all modalities, six factors shared by subsets of modalities, and two factors specific to individual modalities. In the **ai case**, L=4, with two factors shared by all modalities and two modality‐specific factors. In the **pi case**, L=4, with two factors shared by subsets of modalities and two modality‐specific factors. In the **ap case**, L=4, with two factors shared by all modalities and two shared by subsets of modalities. Finally, sGBFA and SBFA are implemented under the true graph ${𝒢}_{2}$. A visualization of the factor loading structures W for each case is provided in Supporting Information: Section 3.

For robust analysis, we further specify four working graphs, all built upon star‐like pathways that are aligned with the structure of W. The star‐like pathway has a center node connected directly to all other nodes, with no additional edges among them. In the proposed simulation setting, each modality has 10 pathways of equal size, where the i‐th pathway consists of features from 1+10(i−1) to 10i. Before we define the working graphs, we shall introduce the concepts of within‐pathway edges (**informative edges**), which connect nodes within the same pathway, and across‐pathway edges (**noisy edges**), which connect nodes from different pathways. ${𝒢}_{0}$ refers to the graph without edges, essentially a trivial case. ${𝒢}_{2}$ only comprises the star‐like pathways, representing the scenario with a complete absence of noisy edges. ${𝒢}_{1}$ is obtained by randomly removing edges from ${𝒢}_{2}$ with a probability of 0.3. It captures partial sparsity structures of the factor loadings, which leads to acquiring less information compared to ${𝒢}_{2}$. In contrast, adding within‐pathway edges with a probability of 0.3 to ${𝒢}_{2}$ gives ${𝒢}_{3}$. Note that ${𝒢}_{2}$ is considered as the true graph, with ${𝒢}_{3}$ heuristically designed to provide additional information. Finally, we define ${𝒢}_{4}$ by adding across‐pathway edges to ${𝒢}_{3}$ with a probability of 0.1, with the aim to evaluate the robustness of the proposed method to the noisy edges. Intuitively, the graphs follows the approximate ordering ${𝒢}_{0}<{𝒢}_{1}∼{𝒢}_{4}<{𝒢}_{2}∼{𝒢}_{3}$ according to the information richness they convey.

In Simulation II, we extend one scenario from Simulation I by incorporating outcome variables. Specifically, we focus on the **ai** case with Gaussian data, consisting of p=500 features, n=400 samples, and L=4 latent factors. We use half of the samples for model training and the remaining 200 samples for testing. This setting is chosen to ensure a fair comparison with sJIVE, as sJIVE is designed for Gaussian data and factors that are either modality‐specific or shared across all modalities. For the proposed sGBFA model, we evaluate performance under the graph structure ${𝒢}_{2}$, representing the true graph without noisy edges. We consider three choices for the number of outcomes: {1,5,10}. Suppose the intercept is 0, and each entry of the regression coefficients $\beta_{j}\in {\mathbb{R}}^{1\times L}$ are generated from the uniform distribution 𝒰(1,2), and the outcomes $y_{j}$ follow the Gaussian distribution $𝒩\left(\beta_{j}Z,\sigma_{y}^{2}\right)$, where the outcome noise variance $\sigma_{y}^{2}$ takes values {1,2,3}. Of note, sJIVE is not designed to handle multiple outcomes. Thus, in the 5‐outcome and 10‐outcome settings, we apply sJIVE to one outcome at a time, using the same set of 5 or 10 outcomes.

For all scenarios, nonzero elements of the factor loading matrix W and the latent factor matrix Z are generated from $𝒩\left(0,1.5^{2}\right)$. Location vector m is 0 throughout the simulation studies. For the Gaussian data, $X^{\left(h\right)}=\mu^{\left(h\right)}+\epsilon^{\left(h\right)}$ with $\epsilon^{\left(h\right)}∼𝒩\left(0,\sigma^{2}\right)$. For the binomial data, $X^{\left(h\right)}$ follows $\mathrm{Bin}\left(n_{j}^{\left(h\right)},p_{\mathrm{ji}}^{\left(h\right)}\right)$ with $p_{\mathrm{ji}}^{\left(h\right)}=1/\left(1+\exp\left(-\mu_{\mathrm{ji}}^{\left(h\right)}\right)\right)$. The number of trials $n_{j}^{\left(h\right)}$ for the j‐th variable is randomly sampled from {1,…,10}. For the binary data, we have $n_{j}^{\left(h\right)}\equiv 1$ for all h and j.

We use the relative reconstruction error (RRE) and mean squared error (MSE) to evaluate the performance of the model in terms of dimension reduction and regression, respectively. Let the superscript “mod” denote matrices that are not associated with the outcome. The RRE is defined as $\mathrm{RRE}=∥{\hat{\mu }}^{\mathrm{mod}}-\mu^{\mathrm{mod}}∥/∥\mu^{\mathrm{mod}}∥$, where ${\hat{\mu }}^{\mathrm{mod}}=\frac{1}{𝒯}\sum_{t=1}^{𝒯}\left(m^{\left(t\right)}1^{⊤}+W^{\left(t\right)}Z^{\left(t\right)}\right)$, and t indexes the iteration in the MCMC chain of length 𝒯. Compared with the traditional reconstruction error $∥{\hat{\mu }}^{\mathrm{mod}}-\mu^{\mathrm{mod}}∥$, the RRE offers a fairer basis for comparison across settings with different data types. The MSE of outcome j is defined on the test dataset as $\frac{1}{n}\sum_{i=1}^{n}{\left(y_{\mathrm{ji}}-{\hat{y}}_{\mathrm{ji}}\right)}^{2}$. The predicted outcomes ${\hat{y}}_{j}$ are calculated as follows. First, we estimate model parameters $\left(\hat{W},{\hat{\beta }}_{j},\hat{m},{\hat{\beta }}_{j0}\right)$ using the training dataset, where $\hat{W}=\frac{1}{𝒯}\sum_{t}W^{\left(t\right)}$, $\hat{m}=\frac{1}{𝒯}\sum_{t}m^{\left(t\right)}$, ${\hat{\beta }}_{j}=\frac{1}{𝒯}\sum_{t}\beta_{j}^{\left(t\right)}$, and ${\hat{\beta }}_{j0}=\frac{1}{𝒯}\sum_{t}\beta_{j0}^{\left(t\right)}$. Next, we obtain the estimated latent factors $\hat{Z}=\frac{1}{𝒯}\sum_{t}Z^{\left(t\right)}$ by applying the proposed sGBFA model (excluding the outcome component) to the test dataset while fixing $W=\hat{W}$ and $m=\hat{m}$. Finally, the predicted outcomes are computed as ${\hat{y}}_{j}={\hat{\beta }}_{j0}+{\hat{\beta }}_{j}\hat{Z}$.

**FIGURE 2:**
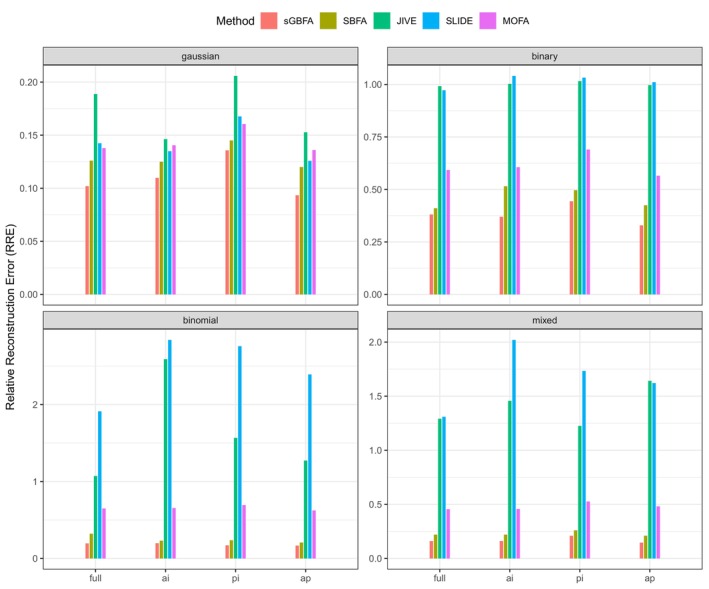
Relative reconstruction error (RRE) of sGBFA and competing methods in Simulation I (p=500, n=200, H=5). Four group structures of the factor loading matrix W (**full**, **ai**, **pi** and **ap**) and four data types (Gaussian, binary, binomial, and mixed) are considered. The results for sGBFA and SBFA are evaluated under ${𝒢}_{2}$, representing the true graph without noisy edges. Results are averaged over 50 repeated samplings.

**FIGURE 3:**
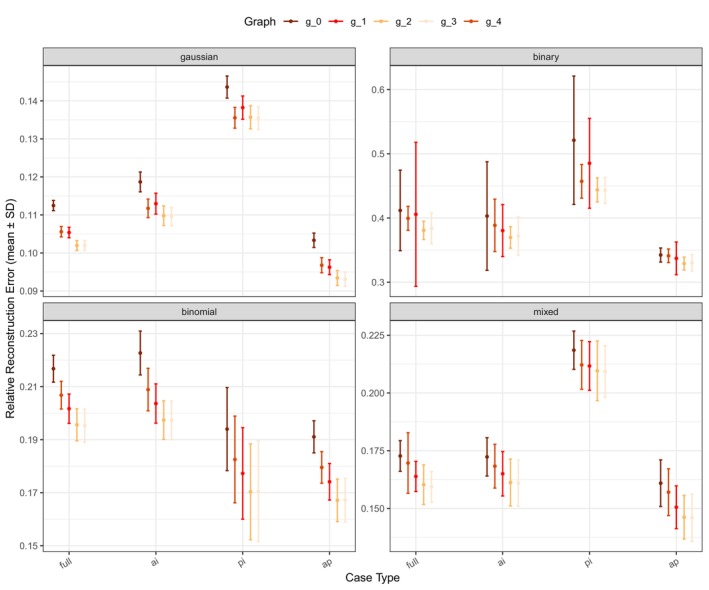
Relative reconstruction error (RRE) with confidence intervals for sGBFA in Simulation I (p=500, n=200, H=5). Four group structures of the factor loading matrix W (**full**, **ai**, **pi** and **ap**), four data types (Gaussian, binary, binomial, and mixed), and four graph structures ${𝒢}_{1}∼{𝒢}_{4}$, along with the null graph ${𝒢}_{0}$, are considered. The graphs follow the approximate ordering ${𝒢}_{0}<{𝒢}_{1}∼{𝒢}_{4}<{𝒢}_{2}∼{𝒢}_{3}$ according to their information richness. Results are averaged over 50 repeated samplings.

For Simulation I, Figure 2 demonstrates that sGBFA consistently outperforms competing methods in terms of RRE across all scenarios. This result highlights both the value of integrating prior graph knowledge and the model's ability to learn various group structures. Existing methods designed primarily for continuous data, such as SLIDE and JIVE, perform poorly on binary, binomial, and mixed data types, underscoring the strength of the proposed unified framework capable of handling heterogeneous data modalities. Furthermore, the comparison between sGBFA and SBFA indicates that the proposed model leverages graph information more effectively. Section 5 further shows that the proposed sGBFA model is robust to misspecified graphs. Although the RRE values under the misspecified graphs ${𝒢}_{1}$ and ${𝒢}_{4}$ are slightly higher than those under ${𝒢}_{2}$ and ${𝒢}_{3}$, they remain better than those obtained under the null graph ${𝒢}_{0}$. When combined with the results in Figure 2, the findings show that even under misspecified graphs, sGBFA outperforms non–graph‐guided methods, reinforcing the advantages and necessity of incorporating graph knowledge. Finally, we found that the observed trend of RRE across the graph configurations ${𝒢}_{0}$ to ${𝒢}_{4}$ aligns with their ordering by information richness: ${𝒢}_{0}<{𝒢}_{1}∼{𝒢}_{4}<{𝒢}_{2}∼{𝒢}_{3}$ (Figure 3).

**FIGURE 4:**
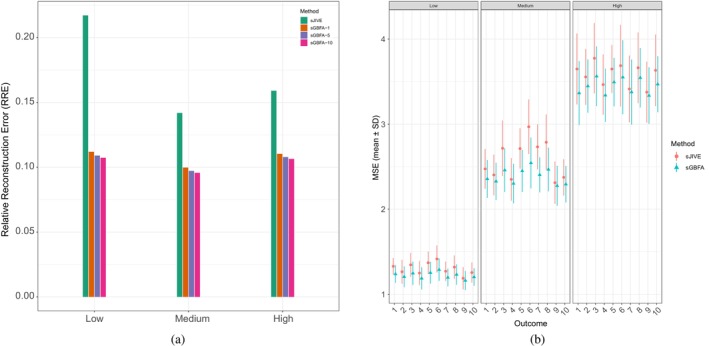
Performance comparison in Simulation II (p=500, n=200, H=5, group structure of W: **ai**, graph structure employed by sGBFA: ${𝒢}_{2}$). For both methods, we consider three noise levels of the outcome Y ∈ {low, medium, high}. Results are averaged over 50 repeated samplings. **(a)**: Comparison of relative reconstruction error (RRE) between sJIVE and sGBFA. The performance of sGBFA is evaluated under three choices for the number of outcomes {1,5,10}, while sJIVE is assessed under the single‐outcome setting. **(b)**: Comparison of mean squared error (MSE) per outcome, with confidence intervals, between sJIVE and sGBFA. The sGBFA model is evaluated under the 10‐outcome setting, whereas sJIVE is assessed under the single‐outcome setting, one outcome at a time, using the same set of outcomes.

Regarding Simulation II, Table 1 shows that sGBFA consistently outperforms sJIVE in terms of MSE across different numbers of outcomes and outcome noise levels. Although the results do not provide clear evidence that using more outcomes directly improves predictive accuracy, we observe that incorporating additional outcomes reduces the standard deviation of the MSE, indicating more stable estimation. Of note, Table 1 reports the average MSE across all outcomes in the multi‐outcome settings. To further evaluate predictive performance at the individual‐outcome level, we compared the MSE for each outcome between sGBFA and sJIVE in the 10‐outcome setting. As shown in Figure 4b, sGBFA yields more accurate predictions than sJIVE for all 10 outcomes. We also examined dimension reduction performance in Simulation II. Beyond exhibiting superior ability to recover the low‐rank representation compared with sJIVE, sGBFA demonstrates an interesting trend: as the number of outcomes increases, its estimated low‐rank representation becomes more accurate according to RRE. This finding suggests that incorporating multiple outcomes may benefit downstream analyses beyond regression.

**TABLE 1:** Comparison of mean squared error (MSE), averaged over outcomes, with standard deviations shown in parentheses for Simulation II (p=500, n=200, H=5, group structure of W: **ai**, graph structure employed by sGBFA: ${𝒢}_{2}$).

# of outcome	Noise level of Y	sGBFA	sJIVE
1	High	3.36 (0.139)	3.65 (0.174)
	Medium	2.36 (0.051)	2.47 (0.054)
	Low	1.24 (0.010)	1.32 (0.010)
5	High	3.44 (0.026)	3.61 (0.131)
	Medium	2.38 (0.011)	2.53 (0.067)
	Low	1.23 (0.004)	1.31 (0.017)
10	High	3.45 (0.010)	3.59 (0.152)
	Medium	2.39 (0.007)	2.58 (0.072)
	Low	1.22 (0.002)	1.30 (0.017)

*Note:* For both methods, we consider three noise levels of the outcome Y ∈ {low, medium, high}. Results are averaged over 50 repeated samplings. The performance of sGBFA is evaluated under three choices for the number of outcomes {1,5,10}, while sJIVE is assessed under the single‐outcome setting, one outcome at a time, using the same set of outcomes.

## 4 Analysis of ADNI Data

AD is an irreversible neurodegenerative disease caused by the deterioration of brain tissue, which results in an accelerated cognitive decline relative to normal aging. While there exist treatments for enhancing impaired neuron systems, these treatments cannot effectively prevent the progression of cognitive decline. Most recent advancements have introduced potential treatments such as aducanumab, lecanemab, and donanemab, which show promise in slowing the progression of AD for certain patients. We obtained the data from the Alzheimer's Disease Neuroimaging Initiative (ADNI), which has generated rich multi‐modal data on genetics, omics, neuroimaging, cognition, and clinical status.

The ADNI dataset that we analyzed includes n=414 subjects, three‐omics modalities (SNP data, metabolomics data and gene expression data), and four outcome variables ranging from cognitive assessments to imaging phenotypes. In particular, we use the following four continuous outcome variables: the MMSE, a validated brief cognitive assessment with scores ranging from 0 to 30; the Clinical Dementia Rating Scale–Sum of Boxes (CDR‐SB) score, a more informational quantitative cognitive index; the fluorodeoxyglucose‐positron emission tomography (FDG‐PET) and 18F‐AV‐45 (AV45), two effective imaging bio‐markers for AD based on PET scans. These outcome variables can measure the progression of AD from different aspects. We perform targeted SNP data analysis based on several key genome‐wide association studies (GWAS) in AD [33, 34], where the top 500 SNPs based on their *p*‐values are included in our analysis. SNP data are viewed as multinomial variables taking genotype values from {0,1,2}, representing the number of minor alleles at the SNP location. For metabolomics data, we have 57 features and the underlying graph is extracted using Mummichog [25]. For gene expression data, we applied the sure independence screening (SIS) [35] to select the most relevant 500 genes from ∼20000 genes, reducing dimensionality from an ultra‐high number of features to a more manageable scale. The response variable used by SIS depends on which outcome we would include in our model. The underlying pathways for gene expression data are extracted from KEGG [24]. Both gene expression and metabolomics data are continuous. For methods that can incorporate outcomes (sGBFA and sJIVE), the models are trained on the training dataset (n=300), while the remained data (n=114) are used for testing the prediction performance (the exact procedure is described in Section 3). For the methods that do not use outcomes, we trained the factor model on the whole dataset (n=414) and estimated the latent factors. Then, the regression model involved with the selected outcome and the estimated latent factors is trained and tested on the same training and test datasets that are used for sGBFA and sJIVE.

Table 2 reports the MSE of predicting different outcomes on the test dataset. sGBFA and SBFA are evaluated either with or without the graph knowledge. As to the incorporation of outcomes, we consider three possible scenarios: no outcome included, one outcome included, and four outcomes included. The results in 2 demonstrate that sGBFA consistently outperforms existing methods in terms of MSE of prediction for different outcomes in the regression model, which reveals the effectiveness of the sGBFA of handling multi‐omics data and incorporation of the graph knowledge. Moreover, including certain outcomes such as MMSE and CDR‐SB improves the prediction performance compared to cases without outcomes.

**TABLE 2:** MSE of sGBFA and existing methods for the ADNI dataset.

Method	Graph	SRL (#)	MMSE	AV45	CDR‐SB	FDG‐PET
sGBFA	✓	✘ (0)	2.770	0.670	0.732	**0.565**
sGBFA	✘	✘ (0)	2.821	0.732	0.821	0.620
sGBFA	✓	✓ (1)	**2.671**	0.653	**0.701**	0.589
sGBFA	✘	✓ (1)	2.763	0.687	0.755	0.655
sGBFA	✓	✓ (4)	2.736	**0.633**	0.730	0.595
sGBFA	✘	✓ (4)	2.785	0.672	0.740	0.647
SBFA	✓	✘ (0)	2.807	0.801	0.815	0.593
SBFA	✘	✘ (0)	2.921	0.815	0.920	0.760
sJIVE	✘	✘ (0)	3.103	0.822	0.980	0.773
sJIVE	✘	✓ (1)	3.123	0.830	0.966	0.757
MOFA	✘	✘ (0)	2.953	0.810	0.975	0.762
SLIDE	✘	✘ (0)	3.233	0.836	0.965	0.781

*Note:* Four cognitive/imaging outcomes are considered: MMSE, AV45, CDR‐SB, and FDG‐PET. “Graph” indicates whether the method incorporates graph knowledge (✓: With graph, ✘: Without graph). “SRL” (simultaneous representation learning) indicates whether the method employs a simultaneous representation learning framework. The number of included outcomes is shown in parentheses. When this value equals 1, the outcome included in the simultaneous representation learning is the same as the outcome being predicted. When equal to 4, all four outcomes are included. The last four columns report MSE for each outcome. The bold values indicate the best performing values of each column.

Additionally, the pathway enrichment analysis, which is conducted through ToppGene Suite and MetaboAnalyst, further supports the biological validity and usefulness of the sGBFA. It demonstrates the effectiveness of the sGBFA to identify meaningful biological pathways that are relevant to the disease of interest. For example, in the scenario with incorporating the graph knowledge and the outcome MMSE, the sGBFA ends up with 15 latent factors in which include 11 latent factors that are modality specific, 3 latent factors that are shared by two modalities and 1 latent factor that is shared by all three modalities. We examined the top five pathways enriched by the selected genes or metabolites (with the smallest *p*‐values) and found that the identified pathways including the mTOR signaling pathway, the MAPK signaling pathway, the TCR signaling in naive CD4+ T cells pathway, and the Aminoacyl‐tRNA biosynthesis pathway, have been shown to be closely related to AD [36, 37, 38, 39]. This affirms that our method can provide important insights about molecular underpinning of AD.

## 5 Discussion

Motivated by the need to understand the complex mechanism of AD, we proposed a supervised graph‐guided factor analysis model for high‐dimensional multi‐omics AD data to identify a biologically meaningful low dimensional representation that can be used to predict imaging phenotypes and clinical phenotypes simultaneously. This method uses novel priors to effectively incorporate the graph knowledge and can handle diverse data types, including categorical, continuous, and count data. Additionally, by learning sparsity at both the feature and modality levels, the model is able to retrieve a more informative low‐dimensional representation. Through extensive simulations, we demonstrate the superior performance of our method in both dimension reduction and outcome prediction and that our model is fairly robust to noisy graphs. Analysis of the ADNI data using our model shows that the estimated factors provide important insights about AD biology.

There are several directions for future research. First, enhancing the computational efficiency of algorithms to explore posteriors is important when dealing with high‐dimensional data. Potential alternatives to the MCMC algorithm include expectation–maximization (EM) and variational Bayesian methods. Second, extending sGBFA into the simultaneous representation learning method that can accommodate other predictive models, such as survival analysis in addition to regression models, would be a valuable avenue for further investigation. Lastly, broadening the scope of the proposed approach to tackle more complicated problems, such as scenarios with complex graph structures, incomplete multi‐omics data, presents another promising direction.

## Funding

This work was supported by National Institutes of Health, RF1AG063481, R01AG071174.

## Conflicts of Interest

The authors declare no conflicts of interest.

## Supporting information


**Data S1:** sim70570‐sup‐0001‐Supinfo.pdf.

## Data Availability

The data that support the findings of this study are openly available in ADNI at https://adni.loni.usc.edu.

## References

[1] M. Olivier , R. Asmis , G. A. Hawkins , T. D. Howard , and L. A. Cox , “The Need for Multi‐Omics Biomarker Signatures in Precision Medicine,” International Journal of Molecular Sciences 20, no. 19 (2019): 4781. 31561483 10.3390/ijms20194781 PMC6801754

[2] Y. Zhou , J. Fang , L. M. Bekris , et al., “AlzGPS: A Genome‐Wide Positioning Systems Platform to Catalyze Multi‐Omics for Alzheimer's Drug Discovery,” Alzheimer's Research & Therapy 13, no. 1 (2021): 1–13. 10.1186/s13195-020-00760-w PMC7804907 33441136

[3] N. Wani and K. Raza , “Integrative Approaches to Reconstruct Regulatory Networks From Multi‐Omics Data: A Review of State‐Of‐The‐Art Methods,” Computational Biology and Chemistry 83 (2019): 107120. 31499298 10.1016/j.compbiolchem.2019.107120

[4] I. Subramanian , S. Verma , S. Kumar , A. Jere , and K. Anamika , “Multi‐Omics Data Integration, Interpretation, and Its Application,” Bioinformatics and Biology Insights 14 (2020): 1177932219899051. 32076369 10.1177/1177932219899051 PMC7003173

[5] O. B. Poirion , Z. Jing , K. Chaudhary , S. Huang , and L. X. Garmire , “DeepProg: An Ensemble of Deep‐Learning and Machine‐Learning Models for Prognosis Prediction Using Multi‐Omics Data,” Genome Medicine 13 (2021): 1–15. 34261540 10.1186/s13073-021-00930-x PMC8281595

[6] R. Shen , A. B. Olshen , and M. Ladanyi , “Integrative Clustering of Multiple Genomic Data Types Using a Joint Latent Variable Model With Application to Breast and Lung Cancer Subtype Analysis,” Bioinformatics 25, no. 22 (2009): 2906–2912. 19759197 10.1093/bioinformatics/btp543 PMC2800366

[7] E. F. Lock , K. A. Hoadley , J. S. Marron , and A. B. Nobel , “Joint and Individual Variation Explained (JIVE) for Integrated Analysis of Multiple Data Types,” Annals of Applied Statistics 7, no. 1 (2013): 523–542. 23745156 10.1214/12-AOAS597 PMC3671601

[8] G. Zhou , A. Cichocki , Y. Zhang , and D. P. Mandic , “Group Component Analysis for Multiblock Data: Common and Individual Feature Extraction,” IEEE Transactions on Neural Networks and Learning Systems 27, no. 11 (2015): 2426–2439. 26529787 10.1109/TNNLS.2015.2487364

[9] Z. Yang and G. Michailidis , “A Non‐Negative Matrix Factorization Method for Detecting Modules in Heterogeneous Omics Multi‐Modal Data,” Bioinformatics 32, no. 1 (2016): 1–8. 26377073 10.1093/bioinformatics/btv544 PMC5006236

[10] I. Gaynanova and G. Li , “Structural Learning and Integrative Decomposition of Multi‐View Data,” Biometrics 75, no. 4 (2019): 1121–1132. 31254385 10.1111/biom.13108

[11] A. Klami , S. Virtanen , E. Leppäaho , and S. Kaski , “Group Factor Analysis,” IEEE Transactions on Neural Networks and Learning Systems 26, no. 9 (2014): 2136–2147. 25532193 10.1109/TNNLS.2014.2376974

[12] S. Zhao , C. Gao , S. Mukherjee , and B. E. Engelhardt , “Bayesian Group Factor Analysis With Structured Sparsity,” Journal of Machine Learning Research 17, no. 1 (2016): 6868–6914. PMC12456737 40994559

[13] E. Leppäaho and S. din A.‐uM, Kaski , “GFA: Exploratory Analysis of Multiple Data Sources With Group Factor Analysis,” Journal of Machine Learning Research 18, no. 1 (2017): 1294–1298.

[14] Q. Mo , R. Shen , C. Guo , M. Vannucci , K. S. Chan , and S. G. Hilsenbeck , “A Fully Bayesian Latent Variable Model for Integrative Clustering Analysis of Multi‐Type Omics Data,” Biostatistics 19, no. 1 (2018): 71–86. 28541380 10.1093/biostatistics/kxx017 PMC6455926

[15] E. J. Min , C. Chang , and Q. Long , Generalized Bayesian Factor Analysis for Integrative Clustering With Applications to Multi‐Omics Data (IEEE, 2018), 109–119. 10.1109/DSAA.2018.00021 PMC6521881 31106307

[16] R. Argelaguet , B. Velten , D. Arnol , et al., “Multi‐Omics Factor Analysis—A Framework for Unsupervised Integration of Multi‐Omics Data Sets,” Molecular Systems Biology 14, no. 6 (2018): e8124. 29925568 10.15252/msb.20178124 PMC6010767

[17] Z. Sun , D. Chung , B. Neelon , et al., “A Bayesian Framework for Pathway‐Guided Identification of Cancer Subgroups by Integrating Multiple Types of Genomic Data,” Statistics in Medicine 42, no. 28 (2023): 5266–5284. 37715500 10.1002/sim.9911 PMC12167630

[18] E. F. Palzer , C. H. Wendt , R. P. Bowler , C. P. Hersh , S. E. Safo , and E. F. Lock , “sJIVE: Supervised Joint and Individual Variation Explained,” Computational Statistics and Data Analysis 175 (2022): 107547. 36119152 10.1016/j.csda.2022.107547 PMC9481062

[19] T. Chekouo and S. E. Safo , “Bayesian Integrative Analysis and Prediction With Application to Atherosclerosis Cardiovascular Disease,” Biostatistics 24, no. 1 (2023): 124–139. 10.1093/biostatistics/kxab016 PMC9960952 33969382

[20] S. Samorodnitsky , C. H. Wendt , and E. F. Lock , “Bayesian Simultaneous Factorization and Prediction Using Multi‐Omic Data,” Computational Statistics and Data Analysis 197 (2024): 107974. 38947282 10.1016/j.csda.2024.107974 PMC11210674

[21] C. Li and H. Li , “Network‐Constrained Regularization and Variable Selection for Analysis of Genomic Data,” Bioinformatics 24, no. 9 (2008): 1175–1182. 18310618 10.1093/bioinformatics/btn081

[22] C. Chang , S. Kundu , and Q. Long , “Scalable Bayesian Variable Selection for Structured High‐Dimensional Data,” Biometrics 74, no. 4 (2018): 1372–1382. 29738602 10.1111/biom.12882 PMC6222001

[23] Y. Zhao , C. Chang , and Q. Long , “Knowledge‐Guided Statistical Learning Methods for Analysis of High‐Dimensional‐Omics Data in Precision Oncology,” JCO Precision Oncology 3 (2019): 1–9. 10.1200/PO.19.00018 PMC9797232 35100722

[24] M. Kanehisa and S. Goto , “KEGG: Kyoto Encyclopedia of Genes and Genomes,” Nucleic Acids Research 28, no. 1 (2000): 27–30. 10592173 10.1093/nar/28.1.27 PMC102409

[25] S. Li , Y. Park , S. Duraisingham , et al., “Predicting Network Activity From High Throughput Metabolomics,” PLoS Computational Biology 9, no. 7 (2013): e1003123. 23861661 10.1371/journal.pcbi.1003123 PMC3701697

[26] V. K. Gore and M. R. Jerrum , “The Swendsen–Wang Process Does Not Always Mix Rapidly,” Journal of Statistical Physics 97, no. 1 (1999): 67–86.

[27] J. Bao , C. Chang , Q. Zhang , A. J. Saykin , L. Shen , and Q. Long , “Integrative Analysis of Multi‐Omics and Imaging Data With Incorporation of Biological Information via Structural Bayesian Factor Analysis,” Briefings in Bioinformatics 24, no. 2 (2023): bbad073. 36882008 10.1093/bib/bbad073 PMC10387302

[28] K. Q. Wenrui Li and Q. Long , “Graph‐Guided Bayesian Factor Model for Integrative Analysis of Multi‐Modal Data With Noisy Network Information,” Statistics in Biosciences (2024): 1–17, 10.1007/s12561-024-09452-7. PMC12221265 40693697

[29] Q. Zhang , C. Chang , L. Shen , and Q. Long , “Incorporating Graph Information in Bayesian Factor Analysis With Robust and Adaptive Shrinkage Priors,” Biometrics 80, no. 1 (2024): ujad014. 38281768 10.1093/biomtc/ujad014 PMC10826885

[30] Y. T. Huang , T. J. VanderWeele , and X. Lin , “Joint Analysis of SNP and Gene Expression Data in Genetic Association Studies of Complex Diseases,” Annals of Applied Statistics 8, no. 1 (2014): 352. 24729824 10.1214/13-AOAS690 PMC3981558

[31] F. Li and N. R. Zhang , “Bayesian Variable Selection in Structured High‐Dimensional Covariate Spaces With Applications in Genomics,” Journal of the American Statistical Association 105, no. 491 (2010): 1202–1214, 10.1198/jasa.2010.tm08177.

[32] A. Gelman , J. B. Carlin , H. S. Stern , D. B. Dunson , A. Vehtari , and D. B. Rubin , Bayesian Data Analysis (CRC press, 2013).

[33] I. E. Jansen , J. E. Savage , K. Watanabe , et al., “Genome‐Wide Meta‐Analysis Identifies New Loci and Functional Pathways Influencing Alzheimer's Disease Risk,” Nature Genetics 51, no. 3 (2019): 404–413. 30617256 10.1038/s41588-018-0311-9 PMC6836675

[34] B. W. Kunkle , B. Grenier‐Boley , R. Sims , et al., “Genetic Meta‐Analysis of Diagnosed Alzheimer's Disease Identifies New Risk Loci and Implicates Aβ, Tau, Immunity and Lipid Processing,” Nature Genetics 51, no. 3 (2019): 414–430. 30820047 10.1038/s41588-019-0358-2 PMC6463297

[35] J. Fan and J. Lv , “Sure Independence Screening for Ultrahigh Dimensional Feature Space,” Journal of the Royal Statistical Society, Series B: Statistical Methodology 70, no. 5 (2008): 849–911. 10.1111/j.1467-9868.2008.00674.x PMC2709408 19603084

[36] S. Oddo , “The Role of mTOR Signaling in Alzheimer Disease,” Frontiers in Bioscience (Scholar Edition) 4 (2012): 941–952. 22202101 10.2741/s310 PMC4111148

[37] L. Munoz and A. J. Ammit , “Targeting p38 MAPK Pathway for the Treatment of Alzheimer's Disease,” Neuropharmacology 58, no. 3 (2010): 561–568. 19951717 10.1016/j.neuropharm.2009.11.010

[38] L. Dai , Q. Wang , X. Lv , F. Gao , Z. Chen , and Y. Shen , “Elevated β‐Secretase 1 Expression Mediates CD4+ T Cell Dysfunction via PGE2 Signalling in Alzheimer's Disease,” Brain, Behavior, and Immunity 98 (2021): 337–348. 34500034 10.1016/j.bbi.2021.08.234

[39] E. Trushina , T. Dutta , X. M. T. Persson , M. M. Mielke , and R. C. Petersen , “Identification of Altered Metabolic Pathways in Plasma and CSF in Mild Cognitive Impairment and Alzheimer's Disease Using Metabolomics,” PLoS One 8, no. 5 (2013): e63644. 23700429 10.1371/journal.pone.0063644 PMC3658985

