# Supplementary material for: Simultaneous Representation Learning of Multi‐Omics and Clinical Outcome Data via a Supervised Knowledge‐Guided Bayesian Factor Model
Source: Stat Med. 2026 Apr 26;45:e70570. doi: 10.1002/sim.70570 (PMC13110451; doi:10.1002/sim.70570)
Supplement: Supplementary file 1 — Data S1: sim70570‐sup‐0001‐Supinfo.pdf. [file SIM-45-0-s001.pdf]

Web Appendix

1 | MCMC ALGORITHM

In this section, we present the detailed derivation of the MCMC algorithm. Section 1.1 discusses two data augmentation techniques utilized by the proposed algorithm. Section 1.2 describes the conditional posterior distribution of each parameter. For the sake of notation convenience, we use the superscript $h = 0$ to index parameters associated with the outcome modality in the supplementary. Specifically, $\boldsymbol{w}_j^{(0)}$ and $\lambda^{(0)}$ are equivalent to $\boldsymbol{\beta}_j$ and λ^y in the main article, respectively. Additionally, we set $\phi_l^{(0)} \equiv 1$ for $1 \leq l \leq L$.

1.1 | Data Augmentation

In the proposed algorithm, we use two data augmentation techniques. First, the augmentation of the Pólya Gamma latent variable ρ_{ji} [1] for the discrete data x_{ji}:

$$\frac{e^{\mu_{ji}x_{ji}}}{(1+e^{\mu_{ji}})^{b_{ji}}} = 2^{-b_{ji}} e^{\kappa_{ji}\mu_{ji}} \int_0^\infty e^{-\rho_{ji}\mu_{ji}^2/2} \pi(\rho_{ji}) d\rho_{ji}, \tag{1}$$

facilitates the unified likelihood framework for heterogeneous data types:

$$\log \pi^{(h)}(\boldsymbol{x}_j^{(h)}, \boldsymbol{\rho}_j^{(h)} | \boldsymbol{\mu}_j^{(h)}) = \log \pi^{(h)}(\boldsymbol{\rho}_j^{(h)}) - \frac{1}{2}\sum_i \rho_{ji}^{(h)}(\mu_{ji}^{(h)} - \psi_{ji}^{(h)})^2 + \sum_i \kappa_{ji}^{(h)}\mu_{ji}^{(h)}, \tag{2}$$

where $\boldsymbol{x}_j^{(h)} = (x_{j1}^{(h)}, \ldots, x_{jn}^{(h)})$, $\boldsymbol{\rho}_j^{(h)} = (\rho_{j1}^{(h)}, \ldots, \rho_{jn}^{(h)})$ and $\boldsymbol{\mu}_j^{(h)} = (\mu_{j1}^{(h)}, \ldots, \mu_{jn}^{(h)})$ refer to the j-th row of $X^{(h)}$, $\rho^{(h)}$, and $\mu^{(h)}$ respectively. The values of $\psi_{ji}^{(h)}$, $\kappa_{ji}^{(h)}$, and $b_{ji}^{(h)}$ and the prior density $\pi^{(h)}(\boldsymbol{\rho}_j^{(h)})$ vary with the type of $\boldsymbol{x}_j^{(h)}$. If $\boldsymbol{x}_j^{(h)}$ follows Gaussian distribution, we have $\psi_{ji}^{(h)} = x_{ji}^{(h)}$, $\kappa_{ji}^{(h)} = 0$, $b_{ji}^{(h)} = \text{NA}$ and $\rho_{ji}^{(h)} \equiv \rho_j^{(h)}$, which refers to the precision parameter with the gamma prior $\mathcal{G}(\frac{\zeta_j}{2}, \frac{\zeta_j}{2})$. If $\boldsymbol{x}_j^{(h)}$ is discrete (binomial or negative binomial), we have $\psi_{ji}^{(h)} = 0$, $\kappa_{ji}^{(h)} = x_{ji}^{(h)} - b_{ji}^{(h)}/2$, and $\pi^{(h)}(\rho_{ji}^{(h)})$ is the density of the Pólya-Gamma distribution $\mathcal{PG}(b_{ji}^{(h)}, 0)$ with $b_{ji}^{(h)} = n_j^{(h)}$ for binomial variables or $b_{ji}^{(h)} = x_{ji}^{(h)} + r_j^{(h)}$ for negative binomial variables. Note that $n_j^{(h)}$ ($r_j^{(h)}$) refer to the number of trials (failures until the experiment is stopped) in binomial distribution (negative binomial distribution). For each h, $\kappa^{(h)}$, $\psi^{(h)}$ and $b^{(h)}$ are the $p_h \times n$ matrices consisting of $\kappa_{ji}^{(h)}$, $\psi_{ji}^{(h)}$ and $b_{ji}^{(h)}$ for $1 < j \leq p_h$ and $1 \leq h \leq H$. Finally, a random variable $\mathcal{X}$ follows the Pólya-Gamma distribution $\mathcal{PG}(b, c)$ with $b > 0$ and $c \in \mathcal{R}$ if

$$\mathcal{X} \overset{D}{=} \frac{1}{2\pi^2} \sum_{k=1}^{\infty} \frac{g_k}{(k-1/2)^2 + c^2/(4\pi^2)}, \tag{3}$$

where $g_k \sim \mathcal{G}(b, 1)$ are independent gamma random variables and $\overset{D}{=}$ refers to equality in distribution.

The second data augmentation technique is to replace the Laplace distribution of $w_{jl}^{(h)}$ with a scale mixture of normals [2]:

$$\frac{a}{2} e^{-a|z|} = \int_0^\infty \frac{\sqrt{a}}{\sqrt{2\pi s}} e^{-az^2/(2s)} \frac{a}{2} e^{-as/2} ds, \quad a > 0, \tag{4}$$

which results in a tractable conditional posterior for $w_{jl}^{(h)}$. In our specifications, let $a = \phi_l^{(h)}\lambda_{jl}^{(h)}$, $z = w_{jl}^{(h)}$ and $s = \tau_{jl}^{(h)}$, the prior for $w_{jl}^{(h)}$ augmented by Equation (4) has the following hierarchical representations:

$$\begin{aligned} w_{jl}^{(h)} | \tau_{jl}^{(h)}, \lambda_{jl}^{(h)}, \phi_l^{(h)} &\sim \mathcal{N}(0, \tau_{jl}^{(h)}/(\phi_l^{(h)}\lambda_{jl}^{(h)})), \\ \tau_{jl}^{(h)} | \lambda_{jl}^{(h)}, \phi_l^{(h)} &\sim \frac{\phi_l^{(h)}\lambda_{jl}^{(h)}}{2} e^{-\frac{\phi_l^{(h)}\lambda_{jl}^{(h)}}{2}\tau_{jl}^{(h)}}, \end{aligned} \tag{5}$$

where $\mathcal{N}(c, d)$ refers to the Gaussian density with mean c and variance (or covariance matrix) d.

1.2 | MCMC

We develop the MCMC algorithm to sample from the following full posterior:

$$\begin{aligned}\pi(W, Z, \boldsymbol{m}, \rho, \lambda, \tau, \phi, \Omega | X) \propto \quad & \pi(X|\boldsymbol{m}, W, Z, \rho)\pi(\boldsymbol{m})\pi(Z)\pi(W|\lambda, \phi, \tau) \\ \times \quad & \pi(\tau|\lambda, \phi)\pi(\phi)\pi(\lambda^{(0)})\pi(\alpha^{\text{mod}}|\Omega)\pi(\Omega)\pi(\rho),\end{aligned} \tag{6}$$

where W includes both factor loading matrix and regression parameters $\boldsymbol{w}_j^{(0)}$ for $1 \leq j \leq p_y$.

The conditional distribution for W and τ: Suppose $\boldsymbol{m}_j^{(h)} = m_j^{(h)}\mathbf{1} \in \mathbb{R}^{n\times 1}$ with $m_j^{(h)}$ being the j-th entry of $\boldsymbol{m}^{(h)}$, $\boldsymbol{\tau}_j^{(h)}$, $\boldsymbol{\kappa}_j^{(h)}$, $\boldsymbol{\lambda}_j^{(h)}$, $\boldsymbol{\rho}_j^{(h)}$ and $\boldsymbol{\psi}_j^{(h)}$ are the j-th row of $\tau^{(h)}$, $\kappa^{(h)}$, $\lambda^{(h)}$, $\rho^{(h)}$ and $\psi^{(h)}$. $\boldsymbol{\phi}^{(h)}$ is the h-row of ϕ. $P_j^{(h)} = \text{diag}(\boldsymbol{\rho}_j^{(h)}) \in \mathbb{R}^{n\times n}$ refers to the diagonal matrix with diagonal entries $\boldsymbol{\rho}_j^{(h)}$. For $h \in \{0, \ldots, H\}$, the conditional posterior distributions for $\boldsymbol{w}_j^{(h)}$ and $\tau_{jl}^{(h)}$ for $1 \leq j \leq p_h$ and $1 \leq l \leq L$ are given by:

$$(\boldsymbol{w}_j^{(h)})^T | \boldsymbol{x}_j^{(h)}, Z, \boldsymbol{m}_j^{(h)}, (\boldsymbol{\tau}_j^{(h)})^2, P_j^{(h)}, \boldsymbol{\phi}^{(h)}, \boldsymbol{\lambda}_j^{(h)} \sim \mathcal{N}(\boldsymbol{a}_j^{(h)}, B_j^{(h)}), \tag{7}$$

$$\frac{1}{\tau_{jl}^{(h)}} | \phi_l^{(h)}, \lambda_{jl}^{(h)} \sim \mathcal{IG}(c_{jl}^{(h)}, d_{jl}^{(h)}), \tag{8}$$

where $\mathcal{IG}(c, d)$ is the inverse-Gaussian distribution with the following density [3]:

$$f(x) = \sqrt{\frac{d}{2\pi}} x^{-3/2} \exp\left\{ -\frac{d(x-c)^2}{2c^2 x} \right\}, \quad x > 0.$$

For Equation (7), we have $\boldsymbol{a}_j^{(h)} = B_j^{(h)} Z P_j^{(h)} (\boldsymbol{\psi}_j^{(h)} - \boldsymbol{m}_j^{(h)} + (P_j^{(h)})^{-1} \boldsymbol{\kappa}_j^{(h)})$ with $B_j^{(h)} = (Z P_j^{(h)} Z^T + (D_{\lambda\phi})_j^{(h)} ((D_\tau)_j^{(h)})^{-1})^{-1}$, $(D_\tau)_j^{(h)} = \text{diag}(\tau_{j1}^{(h)}, \ldots, \tau_{jl}^{(h)}) \in \mathbb{R}^{L\times L}$ and $(D_{\lambda\phi})_j^{(h)} = \text{diag}(\phi_1^{(h)}\lambda_{j1}^{(h)}, \ldots, \phi_L^{(h)}\lambda_{jL}^{(h)}) \in \mathbb{R}^{L\times L}$. For Equation (8), $c_{jl}^{(h)} = \left| \frac{1}{w_{jl}^{(h)}} \right|$ and $d_{jl}^{(h)} = \phi_l^{(h)}\lambda_{jl}^{(h)}$.

The conditional distribution for $\phi_l^{(h)}$: The conditional posterior of $\phi_l^{(h)}$ for $1 \leq l \leq L$ and $1 \leq h \leq H$ is given by:

$$\phi_l^{(h)} | \tau_{jl}^{(h)}, \lambda_{jl}^{(h)} \sim \mathcal{G}\left(a_\phi + \frac{3}{2} p_h, b_\phi + \frac{\sum_j \tau_{jl}^{(h)} \lambda_{jl}^{(h)}}{2} + \sum_j \frac{\lambda_{jl}^{(h)} (w_{jl}^{(h)})^2}{2\tau_{jl}^{(h)}} \right), \tag{9}$$

where $\mathcal{G}(a, b)$ refers to the gamma distribution with the shape parameter a and rate parameter b.

The conditional distribution for λ: The conditional posterior of λ has two parts, which are given by:

$$\lambda_{jl}^{(0)} | w_{jl}^{(0)}, \tau_{jl}^{(0)} \sim \mathcal{G}\left(a_\lambda + \frac{3}{2}, b_\lambda + \frac{\tau_{jl}^{(0)}}{2} + \frac{(w_{jl}^{(0)})^2}{2\tau_{jl}^{(0)}} \right), \tag{10}$$

$$\begin{aligned}\boldsymbol{\alpha}_l^{\text{mod}} | \Omega, \tau, W, \phi \propto\ & e^{-\frac{1}{2\nu_2}(\boldsymbol{\alpha}_l^{\text{mod}} - \nu_1 \mathbf{1})^T \Omega (\boldsymbol{\alpha}_l^{\text{mod}} - \nu_1 \mathbf{1})} \\ & \times \prod_h \prod_j (\lambda_{jl}^{(h)})^{3/2} e^{-\left(\tau_{jl}^{(h)}\phi_l^{(h)} + \frac{(w_{jl}^{(h)})^2 \phi_l^{(h)}}{\tau_{jl}^{(h)}}\right)\lambda_{jl}^{(h)}/2},\end{aligned} \tag{11}$$

for $1 \leq j \leq p_0$ and $1 \leq l \leq L$. While $\lambda_{jl}^{(0)}$ can be generated with the Gibbs sampler, we have to use the MH algorithm to sample $\boldsymbol{\alpha}_l^{\text{mod}}$. Noting that the high dimension will cause slow movements of Markov chain [4], extremely deteriorating the performance of the MH algorithm, an alternative strategy is to perform a coordinate-wise MH algorithm. In particular, we sample $\alpha_{jl}^{(h)}$ conditioned on $\boldsymbol{\alpha}_{-j,l}^{\text{mod}}$, the remaining elements of $\boldsymbol{\alpha}_l^{\text{mod}}$ after removing $\alpha_{jl}^{(h)}$. Equation (11) is boiled down to

$$f(\alpha_{jl}^{(h)}) = (\lambda_{jl}^{(h)})^{3/2} e^{-\frac{\lambda_{jl}^{(h)}}{2}\left(\tau_{jl}^{(h)}\phi_l^{(h)} + \frac{(w_{jl}^{(h)})^2 \phi_l^{(h)}}{\tau_{jl}^{(h)}}\right) - \frac{1}{2\nu_2}(\boldsymbol{\alpha}_l^{\text{mod}} - \nu_1\mathbf{1})^T \Omega (\boldsymbol{\alpha}_l^{\text{mod}} - \nu_1 \mathbf{1})}, \tag{12}$$

for $1 \leq j \leq p_h$ and $1 \leq l \leq L$ given $h \in \{1, \ldots, H\}$. Let $\alpha_{jl}^{(h).t-1}$ be the current state of $\alpha_{jl}^{(h)}$. We generate the candidate $\alpha_{jl}^{(h).\text{cand}}$ from the proposal distribution $\mathcal{N}(\alpha_{jl}^{(h).t-1}, q)$ where q is the pre-specified variance that would impact the acceptance rate. The

candidate $\alpha_{jl}^{(h).\text{cand}}$ will be accepted with probability

$$\iota_{jl} = \text{argmin}\left(1, \frac{f(\alpha_{jl}^{(h).\text{cand}})}{f(\alpha_{jl}^{(h).t-1})}\right). \tag{13}$$

The conditional distribution for Ω: Ω serves as a vital role to incorporate the biological graph information, and it is only associated with $X^{(h)}$ for $1 \leq h \leq H$. For the sake of brevity, we omit the superscript h of parameters with subscript j in the following derivations. There are two noteworthy points about sampling Ω. First, Ω is sampled column by column via the block-wise Gibbs sampler in the symmetric updating fashion (update j-th column and j-th row of Ω simultaneously). $\boldsymbol{\omega}_j$, the j-th column of Ω, is divided into the diagonal element and the non-zero off-diagonal elements that are consistent with the underlying graph. Two implicit permutations are performed to obtain the desired block representation of Ω. The first permutation gives us $\Omega = \begin{bmatrix} \Omega_{-j,-j} & \boldsymbol{\omega}_{-j,j} \\ \boldsymbol{\omega}_{-j,j}^T & \omega_{jj} \end{bmatrix}$ with the j-th diagonal element ω_{jj}, the j-th column off-diagonal elements $\boldsymbol{\omega}_{-j,j}$ and the submatrix $\Omega_{-j,-j}$ obtained by removing the j-th row and j-th column from Ω. Secondly, we permute $\boldsymbol{\omega}_{-j,j}$ into $\boldsymbol{\omega}_{-j,j} = (\boldsymbol{\omega}_j^1, \boldsymbol{\omega}_j^0)$, where $\boldsymbol{\omega}_j^1$ and $\boldsymbol{\omega}_j^0$ are nonzero and zero subvectors of $\boldsymbol{\omega}_{-j,j}$. Correspondingly, we have $\Omega_{-j,-j} = \begin{bmatrix} \widetilde{\Omega}_j^{11} & \widetilde{\Omega}_j^{10} \\ \widetilde{\Omega}_j^{01} & \widetilde{\Omega}_j^{00} \end{bmatrix}$ with $\widetilde{\Omega}_j^{10} = (\widetilde{\Omega}_j^{01})^T$. $\Sigma = \Omega^{-1}$ and $A = \eta(\mathbf{1}\mathbf{1}^T + \epsilon I) + \frac{1}{\nu_2}\sum_l(\boldsymbol{\alpha}_l^{\text{mod}} - \nu_1\mathbf{1})(\boldsymbol{\alpha}_l^{\text{mod}} - \nu_1\mathbf{1})^T$ have the similar block representations based on these permutations:

$$\Sigma = \begin{bmatrix} \Sigma_{-j,-j} & \boldsymbol{\sigma}_{-j,j} \\ \boldsymbol{\sigma}_{-j,j}^T & \sigma_{jj} \end{bmatrix}, \Sigma_{-j,-j} = \begin{bmatrix} \widetilde{\Sigma}_j^{11} & \widetilde{\Sigma}_j^{10} \\ \widetilde{\Sigma}_j^{01} & \widetilde{\Sigma}_j^{00} \end{bmatrix}, A = \begin{bmatrix} A_{-j,-j} & \boldsymbol{a}_{-j,j} \\ \boldsymbol{a}_{-j,j}^T & a_{jj} \end{bmatrix}, \boldsymbol{\sigma}_j^1, \boldsymbol{\sigma}_j^0, \boldsymbol{a}_j^1, \boldsymbol{a}_j^0.$$

The conditional posterior of $\boldsymbol{\omega}_j$ with the reparameterization and the constraint to the underlying graph can be factorized into two independent variables $\boldsymbol{\omega}_j^1$ and ξ_j:

$$\boldsymbol{\omega}_j^1 | \Omega_{-j,-j}, \alpha^{\text{mod}} \sim \mathcal{N}(\overline{\mu}_j, \overline{\Omega}_j / a_{jj}), \tag{14}$$

$$\xi_j | \Omega_{-j,-j}, \alpha^{\text{mod}} \sim \mathcal{G}\left(\frac{\eta(1+\epsilon) + L}{2} + 1, \frac{1}{2}a_{jj}\right), \tag{15}$$

where $\overline{\mu}_j = -\overline{\Omega}_j \boldsymbol{a}_j^1 / a_{jj}$, $\overline{\Omega}_j = \widetilde{\Omega}_j^{11} - \widetilde{\Omega}_j^{10}(\widetilde{\Omega}_j^{00})^{-1}\widetilde{\Omega}_j^{01}$ and $\xi_j = \omega_{jj} - (\boldsymbol{\omega}_j^1)^T \overline{\Omega}_j^{-1} \boldsymbol{\omega}_j^1$. As a result, ω_{jj} is updated by $\xi_j + (\boldsymbol{\omega}_j^1)^T \overline{\Omega}_j^{-1} \boldsymbol{\omega}_j^1$.

Secondly, the involvement of Σ significantly reduces the computation complexity of our algorithm. We speed up the algorithm based on the following relationship between Σ and Ω:

$$\Sigma_{-j,-j} - \boldsymbol{\sigma}_{-j,j}\boldsymbol{\sigma}_{-j,j}^T / \sigma_{jj} = \Omega_{-j,-j}^{-1} = \begin{bmatrix} \overline{\Omega}_j^{-1} & * \\ * & * \end{bmatrix} \text{ and } \overline{\Omega}_j^{-1} = \widetilde{\Sigma}_j^{11} - \boldsymbol{\sigma}_j^1(\boldsymbol{\sigma}_j^1)^T / \sigma_{jj}.$$

Sampling of $\boldsymbol{\omega}_j$ ends up with updating Σ as followed:

$$\sigma_{jj} \leftarrow \xi_j^{-1}, \tag{16}$$

$$\Sigma_{-j,-j} \leftarrow \Omega_{-j,-j}^{-1} + \sigma_{jj}\Omega_{-j,-j}^{-1}\boldsymbol{\omega}_{-j,j}\boldsymbol{\omega}_{-j,j}^T\Omega_{-j,-j}^{-1}, \tag{17}$$

$$\boldsymbol{\sigma}_{-j,j} \leftarrow -\Sigma_{-j,-j}\boldsymbol{\omega}_{-j,j} / \omega_{jj}, \tag{18}$$

The conditional distribution for Z: The conditional posterior distribution of the i-th column $\widetilde{z}_i$ of Z for $i = 1, \ldots, n$ follows the multivariate Gaussian distribution. Let $\widetilde{\boldsymbol{x}}_i$, $\widetilde{\boldsymbol{\kappa}}_i$, $\widetilde{\boldsymbol{\rho}}_i$ and $\widetilde{\boldsymbol{\psi}}_i$ be the i-th column of X, κ, ρ and ψ respectively, where ρ, κ and ψ are concatenated by $\rho^{(h)}$, $\kappa^{(h)}$ and $\psi^{(h)}$ for $0 \leq h \leq H$ in the same fashion of X. $\widetilde{P}_i = \text{diag}(\widetilde{\boldsymbol{\rho}}_i) \in \mathbb{R}^{p \times p}$ refers to the diagonal matrix with $\widetilde{\boldsymbol{\rho}}_i$ as diagonal entries. The posterior distribution of $\widetilde{z}_i$ is given by:

$$\widetilde{z}_i | \widetilde{\boldsymbol{x}}_i, W, \boldsymbol{m}, \widetilde{P}_i \sim \mathcal{N}((W^T\widetilde{P}_iW + I)^{-1}W^T(\widetilde{P}_i(\widetilde{\boldsymbol{\psi}}_i - \boldsymbol{m}) + \widetilde{\boldsymbol{\kappa}}_i), (W^T\widetilde{P}_iW + I)^{-1}). \tag{19}$$

The conditional distribution for $\boldsymbol{m}$: The posterior of $\boldsymbol{m}$ is multivariate Gaussian. $m_j^{(h)}$ is conditionally independent for $1 \leq j \leq p_h$ and $0 \leq h \leq H$, and we have the following conditional posterior for $m_j^{(h)}$:

$$m_j^{(h)} | X, W, Z, \rho \sim \mathcal{N}\left((\varsigma_j^{(h)}(\sum_i \kappa_{ji}^{(h)} - \rho_{ji}^{(h)}((\boldsymbol{w}_j^{(h)})^T\widetilde{z}_i - \psi_{ji}^{(h)}))), \varsigma_j^{(h)}\right), \tag{20}$$

where $\varsigma_j^{(h)} = (\frac{1}{\sigma_m^2} + \sum_i \rho_{ji}^{(h)})^{-1}$ is the posterior variance of $m_j^{(h)}$.

The conditional distribution for $\rho_{ji}^{(h)}$: $\rho_{ji}^{(h)}$ has the conjugate prior regardless of the data type of $x_{ji}^{(h)}$ and the conditional posterior of $\rho_{ji}^{(h)}$ is given by:

$$\rho_{ji}^{(h)} \begin{cases} \sim \mathcal{PG}(b_{ji}^{(h)}, \mu_{ji}^{(h)}), & x_{ji}^{(h)} \text{ is discrete,} \quad (21a) \\ \equiv \rho_j^{(h)} \sim \mathcal{G}\left(\dfrac{\zeta_j + n}{2}, \dfrac{\zeta_j + \sum_i (x_{ji}^{(h)} - \mu_{ji}^{(h)})^2}{2}\right), & x_{ji}^{(h)} \text{ is Gaussian,} \quad (21b) \end{cases}$$

where the posterior of $\rho_{ji}^{(h)}$ follows the Pólya-Gamma distribution (3) for the discrete data or the gamma distribution for the Gaussian data.

2 | ADDITIONAL SIMULATION STUDY

2.1 | Simulations with Noise Levels

In this section, we use the superscript 'mod' to denote the matrices/vectors that are not relevant to the outcome modality. For example, μ^{mod} is the row-wise concatenation of $\mu^{(h)}$ for $1 \leq h \leq H$, which is only associated with the factor analysis. The same way of concatenation can be applied to W^{mod}, $\boldsymbol{m}^{\text{mod}}$ and their estimates. The parameters without the superscript 'mod' relate to all of modalities.

In the simulation study without outcomes, we consider $H = 3$ modalities with sample size $n = 100$, for the low dimensional setting $p_1 = p_2 = p_3 = 30$ and the high dimensional setting $p_1 = 90, p_2 = 60, p_3 = 30$. In the block representation Equation (22) of the factor loading matrix, let $W^{\text{mod}}[, i]$ be the i-th block column of W^{mod} and $W^{\text{mod}}[, i : j]$ for $i < j$ be the submatrix consisting of the block columns from i to j. Three latent factors are associated with the first block-column $W^{\text{mod}}[, 1]$, implying $W_{123}^{(h)} \in \mathbb{R}^{p_h \times 3}$ for $h = 1, 2, 3$, while all the other block-columns are connected to one latent factor. We consider four cases whose factor loading matrices are different combinations of block-columns from the representation 22. Specifically, $W^{\text{mod}}[, 1] \in \mathbb{R}^{p \times 3}$ with $L = 3$, $W^{\text{mod}}[, 1 : 4] \in \mathbb{R}^{p \times 6}$ with $L = 6$, $W^{\text{mod}}[, 5 : 7] \in \mathbb{R}^{p \times 3}$ with $L = 3$ and $W^{\text{mod}} \in \mathbb{R}^{p \times 9}$ with $L = 9$ are considered as the factor loading matrix by Case I, Case II, Case III and Case IV respectively. Figure 1 illustrates the sparse structure of W^{mod} (Figure 1a for the low dimensional setting, Figure 1b for the high dimensional setting).

$$W^{\text{mod}} = \begin{pmatrix} W_{123}^{(1)} & W_{12}^{(1)} & W_{13}^{(1)} & 0 & W_1^{(1)} & 0 & 0 \\ W_{123}^{(2)} & W_{12}^{(2)} & 0 & W_{23}^{(2)} & 0 & W_2^{(2)} & 0 \\ W_{123}^{(3)} & 0 & W_{13}^{(3)} & W_{23}^{(3)} & 0 & 0 & W_3^{(3)} \end{pmatrix}, \quad (22)$$

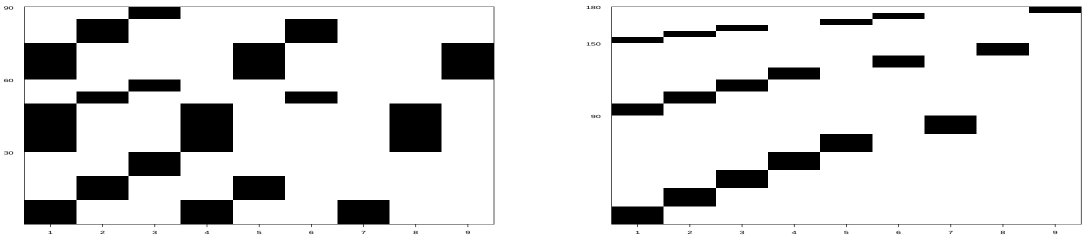

(a) (b)

F I G U R E 1: Sparsity structures of the factor loading matrix with Figure 1a (Figure 1b) corresponding to the low (high) dimensional setting. Black blocks are non-zero parts.

For all scenarios, non-zero elements of the factor loading matrix and the latent factor matrix are generated from $\mathcal{N}(0, 1.5^2)$. Location vector $\boldsymbol{m}$ is 0 throughout the simulation studies. For the Gaussian data, $X^{(h)} = \mu^{(h)} + \epsilon^{(h)}$ with $\epsilon^{(h)} \sim \mathcal{N}(0, \sigma^2)$. For the binomial data, $X^{(h)}$ follows $\text{Bin}(n_j^{(h)}, p_{ji}^{(h)})$ with $p_{ji}^{(h)} = 1/(1 + \exp(-\mu_{ji}^{(h)}))$. The number of trials $n_j^{(h)}$ for the j-th variable is randomly sampled from $\{1, \ldots, K\}$. Note that setting $n_j^{(h)} \equiv 1$ for all h and j gives the Bernoulli data. The mixed data consist of the Gaussian data (modality 1), the Bernoulli data (modality 2) and the binomial data (modality 3). Furthermore, three noise

levels are considered for the Gaussian and binomial data. Especially, $\sigma \in \{0.1, 0.5, 1.5\}$ and $K \in \{5, 10, 20\}$ control the noise level for the Gaussian and binomial data.

We consider four working graphs, all built upon star-like pathways that are associated with the structure of W. The star-like pathway has a center node connected directly to all other surrounding nodes, with no other edges present. For example, consider the star-like pathways in the low-dimensional setting for three modalities, as indicated by the structure of W (Figure 1a). The sizes of these pathways are as follows: $10, 10, 10$ for modality 1, $20, 5, 5$ for modality 2, and $15, 10, 5$ for modality 3. Before we delineate the working graphs, we shall introduce the concepts of within-pathway edges (informative edges), which connect nodes within the same pathway, and across-pathway edges (noisy edges), which connect nodes from different pathways. $\mathcal{G}_0$ refers to the graph without edges, essentially a trivial case. $\mathcal{G}_2$ only comprises the star-like pathways, representing the idealized scenario with a complete absence of noisy edges. $\mathcal{G}_1$ is obtained by randomly removing edges from $\mathcal{G}_2$ with a probability of 0.3. It captures partial sparsity structures of the factor loadings, which leads to acquiring less information compared to $\mathcal{G}_2$. In contrast, adding within-pathway edges with a probability of 0.3 to $\mathcal{G}_2$ gives $\mathcal{G}_3$. Note that $\mathcal{G}_2$ is considered as the true graph, with $\mathcal{G}_3$ heuristically designed to provide additional information. Finally, we define $\mathcal{G}_3^*$ by adding across-pathway edges to $\mathcal{G}_3$ with a probability of 0.1, with the aim to evaluate the robustness of the proposed method to the noisy edges.

Table 1 (low-dimensional setting) and Table 2 (high-dimensional setting) demonstrate that s-GBFA, with graph incorporation, consistently outperforms its competitors in terms of RRE across all scenarios where outcomes are not included. This highlights the effectiveness of utilizing graph knowledge and the unified framework for handling different data types. Notably, while the performance of s-GBFA under $\mathcal{G}_1$ and $\mathcal{G}_3^*$ is slightly lower than under $\mathcal{G}_2$ and $\mathcal{G}_3$, it remains superior to other non-graph-guided methods, underscoring the benefits and necessity of incorporating graphs. We also compared the performance of s-GBFA and SBFA across various graph structures and found that s-GBFA more effectively leverages graph information. For example, s-GBFA is more robust than SBFA to both false negatives (in $\mathcal{G}_1$) and false positives (in $\mathcal{G}_3^*$) caused by noisy edges. Furthermore, the performance improvement of $\mathcal{G}_3$ over $\mathcal{G}_2$ is more pronounced for s-GBFA, indicating its capability and flexibility in employing different informative graphs. Finally, existing methods designed for continuous data perform poorly on binary, binomial, and mixed data types.

2.2 | Case Study II

In this case study, we compare the s-GBFA and RABFA for the Gaussian data under the Case I ($L = 3$) and Case IV ($L = 9$) in the high dimensional setting. Similar to the setting in the main paper, we have $n = 200$ samples with 100 samples for training and 100 samples for testing, three modalities with $p_1 = 90, p_2 = 60, p_3 = 30$. The graph $\mathcal{G}_2$ is used. The factor loading matrix, latent factors and $X^{(h)}$ for $h = 1, 2, 3$ are generated in the same way as we have done in the previous simulation setting. In particular, $X^{(h)}$ for $h = 1, 2, 3$ is generated at the large noisy level with $\sigma = 1.5$. For s-GBFA, we include 5 outcomes whose regression coefficients follows the uniform distribution $\mathcal{U}(1, 5)$. The outcomes $\boldsymbol{y}$ is generated through the Gaussian distribution $\mathcal{N}(\beta Z, \sigma^2)$, where σ is determined by the signal-to-noise ratio $\|\beta\|^2 / (n(\sigma)^2)$ with $\|\cdot\|$ referring to the Frobenius norm. We consider signal-to-noise ratios 1. The simulation results are based on 20 repeated samplings.

As shown in Table 3, the superior performance of s-GBFA in terms of relative reconstructive error (RRE) and mean square error (MSE) highlights the advantages of the proposed simultaneous representation learning framework. Specifically, s-GBFA is able to recover a more accurate low-rank representation of the high-dimensional data, leading to predictions with smaller errors. Moreover, the improvement of s-GBFA over RABFA is particularly notable in Case IV, emphasizing the importance of modality-level shrinkage.

3 | VISUALIZATION OF SIMULATION SETTING

In this section, Figure 2 visualized the factor loading matrix structures and graphs used in the main article for i) factor loading matrix W under cases **ai**, **ap** and **pi**; ii) three graph structures of a single modality$\mathcal{G}_1$, $\mathcal{G}_3$ and $\mathcal{G}_4$; and iii) three graph structures of five modalities $\mathcal{G}_1$, $\mathcal{G}_3$ and $\mathcal{G}_4$. Note that the case **full** and $\mathcal{G}_2$ have already been visualized in the main article.

REFERENCES

1. Polson NG, Scott JG, Windle J. Bayesian inference for logistic models using Pólya–Gamma latent variables. *Journal of the American statistical Association.* 2013;108(504):1339–1349.

2. Andrews DF, Mallows CL. Scale mixtures of normal distributions. *Journal of the Royal Statistical Society: Series B (Methodological).* 1974;36(1):99–102.
3. Chhikara R. *The Inverse Gaussian Distribution: Theory: Methodology, and Applications*. 95. CRC Press, 1988.
4. Au SK, Beck JL. Estimation of small failure probabilities in high dimensions by subset simulation. *Probabilistic engineering mechanics.* 2001;16(4):263–277.

case	method	Gaussian			binary	binomial			mix
		large	medium	small	-	large	medium	small	-
I	s-GBFA($\mathcal{G}_0$)	0.1333	0.1110	0.0973	0.3412	0.2074	0.2340	0.2757	0.2246
	s-GBFA($\mathcal{G}_1$)	0.1213	0.1007	0.0933	0.3235	0.1912	0.2220	0.2525	0.2117
	s-GBFA($\mathcal{G}_2$)	0.1167	0.0987	0.0872	0.3147	0.1835	0.2186	0.2435	**0.2101**
	s-GBFA($\mathcal{G}_3$)	**0.1150**	**0.0965**	**0.0870**	**0.3100**	**0.1820**	**0.2175**	**0.2421**	0.2101
	s-GBFA($\mathcal{G}_3^*$)	0.1232	0.0996	0.0930	0.3282	0.2025	0.2205	0.2500	0.2109
	SBFA($\mathcal{G}_0$)	0.1335	0.1095	0.0975	0.3455	0.2070	0.2344	0.2763	0.2243
	SBFA($\mathcal{G}_1$)	0.1285	0.1020	0.0952	0.3310	0.2033	0.2276	0.2622	0.2145
	SBFA($\mathcal{G}_2$)	0.1250	0.1010	0.0912	0.3285	0.1967	0.2233	0.2534	0.2123
	SBFA($\mathcal{G}_3$)	0.1250	0.1010	0.0910	0.3272	0.1966	0.2225	0.2537	0.2114
	SBFA($\mathcal{G}_3^*$)	0.1300	0.1054	0.0947	0.3341	0.2051	0.2257	0.2601	0.2150
	JIVE	0.1320	0.1101	0.0977	0.8834	1.3412	1.1132	0.9634	0.9124
	SLIDE	0.1313	0.1120	0.0977	0.8971	1.3121	1.1178	0.9525	0.9199
	MOFA	0.1320	0.1100	0.0971	0.4542	1.3530	1.1322	0.9509	0.6344
II	s-GBFA($\mathcal{G}_0$)	0.1355	0.1175	0.0988	0.3464	0.2130	0.2382	0.2803	0.2311
	s-GBFA($\mathcal{G}_1$)	0.1234	0.1015	0.0933	0.3312	0.2021	0.2311	0.2571	0.2235
	s-GBFA($\mathcal{G}_2$)	0.1165	0.0984	0.0879	0.3185	0.1921	0.2210	0.2432	0.2175
	s-GBFA($\mathcal{G}_3$)	**0.1099**	**0.0951**	**0.0875**	**0.3121**	**0.1830**	**0.2195**	**0.2424**	**0.2166**
	s-GBFA($\mathcal{G}_3^*$)	0.1219	0.1003	0.0932	0.3280	0.2011	0.2235	0.2592	0.2258
	SBFA($\mathcal{G}_0$)	0.1357	0.1183	0.0996	0.3477	0.2198	0.2477	0.2929	0.2386
	SBFA($\mathcal{G}_1$)	0.1287	0.1124	0.0945	0.3421	0.2100	0.2432	0.2869	0.2341
	SBFA($\mathcal{G}_2$)	0.1235	0.1031	0.0889	0.3401	0.2077	0.2333	0.2800	0.2278
	SBFA($\mathcal{G}_3$)	0.1233	0.1017	0.0889	0.3396	0.2075	0.2335	0.2801	0.2260
	SBFA($\mathcal{G}_3^*$)	0.1270	0.1080	0.0937	0.3425	0.2099	0.2375	0.2845	0.2309
	JIVE	0.1347	0.1165	0.0960	0.8723	1.3232	1.1367	0.9500	0.9231
	SLIDE	0.1320	0.1107	0.0950	0.8821	1.3455	1.1380	0.9877	0.9103
	MOFA	0.1357	0.1187	0.0980	0.4656	1.3400	1.1108	0.9865	0.6321
III	s-GBFA($\mathcal{G}_0$)	0.1342	0.1177	0.0979	0.3371	0.2132	0.2451	0.2998	0.2323
	s-GBFA($\mathcal{G}_1$)	0.1215	0.1097	0.0946	0.3321	0.2085	0.2359	0.2572	0.2271
	s-GBFA($\mathcal{G}_2$)	0.1173	0.0978	0.0880	0.3211	0.2020	0.2235	0.2447	**0.2129**
	s-GBFA($\mathcal{G}_3$)	**0.1152**	**0.0899**	**0.0865**	**0.3120**	**0.1832**	**0.2200**	**0.2435**	0.2132
	s-GBFA($\mathcal{G}_3^*$)	0.1233	0.1014	0.0924	0.3301	0.2020	0.2391	0.2477	0.2272
	SBFA($\mathcal{G}_0$)	0.1373	0.1170	0.0977	0.3401	0.2147	0.2485	0.3030	0.2388
	SBFA($\mathcal{G}_1$)	0.1312	0.1105	0.0963	0.3375	0.2104	0.2444	0.2727	0.2332
	SBFA($\mathcal{G}_2$)	0.1255	0.0987	0.0910	0.3250	0.2066	0.2401	0.2633	0.2291
	SBFA($\mathcal{G}_3$)	0.1250	0.0955	0.0903	0.3245	0.2062	0.2388	0.2650	0.2180
	SBFA($\mathcal{G}_3^*$)	0.1285	0.0990	0.0943	0.3330	0.2078	0.2421	0.2689	0.2319
	JIVE	0.1350	0.1164	0.0980	0.8881	1.3530	1.1230	1.002	0.9320
	SLIDE	0.1321	0.1100	0.0938	0.8989	1.3449	1.1200	0.9787	0.9211
	MOFA	0.1330	0.1182	0.0988	0.4420	1.3441	1.1100	0.9725	0.6299
IV	s-GBFA($\mathcal{G}_0$)	0.1323	0.1143	0.0936	0.3385	0.2150	0.2391	0.2804	0.2276
	s-GBFA($\mathcal{G}_1$)	0.1251	0.1032	0.0905	0.3300	0.2021	0.2323	0.2520	0.2223
	s-GBFA($\mathcal{G}_2$)	0.1115	0.0977	0.0834	0.3156	0.1918	0.2270	0.2444	**0.2168**
	s-GBFA($\mathcal{G}_3$)	**0.1071**	**0.0880**	**0.0824**	**0.3122**	**0.1848**	**0.2240**	**0.2357**	0.2170
	s-GBFA($\mathcal{G}_3^*$)	0.1230	0.1110	0.0963	0.3301	0.1993	0.2283	0.2509	0.2241
	SBFA($\mathcal{G}_0$)	0.1334	0.1140	0.0945	0.3401	0.2155	0.2445	0.2810	0.2345
	SBFA($\mathcal{G}_1$)	0.1277	0.1087	0.0923	0.3351	0.2100	0.2377	0.2691	0.2263
	SBFA($\mathcal{G}_2$)	0.1230	0.1021	0.0887	0.3304	0.2033	0.2329	0.2630	0.2201
	SBFA($\mathcal{G}_3$)	0.1230	0.1010	0.0876	0.3312	0.2030	0.2332	0.2624	0.2188
	SBFA($\mathcal{G}_3^*$)	0.1267	0.1044	0.0931	0.3337	0.2076	0.2350	0.2700	0.2243
	JIVE	0.1331	0.1105	0.0935	0.8776	1.3219	1.1208	0.9760	0.9024
	SLIDE	0.1344	0.1121	0.0950	0.8790	1.3209	1.1301	0.9880	0.9078
	MOFA	0.1321	0.1123	0.0930	0.4387	1.3541	1.1320	0.9776	0.6401

TABLE 1. The relative RE of the factor model for the s-GBFA and existing methods in the low dimensional setting ($p_1 = p_2 = p_3 = 30$, n=100). Four structures of W (Case I $\sim$ Case IV), three noise levels (large, medium and small), five graph structures ($\mathcal{G}_0 \sim \mathcal{G}_3^*$) and different data types are considered.

case	method	Gaussian			binary	binomial			mix
		large	medium	small	-	large	medium	small	-
I	s-GBFA($\mathcal{G}_0$)	0.1544	0.1335	0.1112	0.3876	0.2330	0.2994	0.3433	0.3041
	s-GBFA($\mathcal{G}_1$)	0.1444	0.1275	0.1032	0.3727	0.2123	0.2844	0.3295	0.2814
	s-GBFA($\mathcal{G}_2$)	0.1335	**0.1230**	**0.0993**	0.3665	0.2013	0.2633	0.3094	0.2755
	s-GBFA($\mathcal{G}_3$)	**0.1330**	0.1230	0.0994	**0.3613**	**0.2002**	**0.2612**	**0.3052**	**0.2730**
	s-GBFA($\mathcal{G}_3^*$)	0.1352	0.1246	0.0997	0.3722	0.2085	0.2712	0.3231	0.2820
	SBFA($\mathcal{G}_0$)	0.1563	0.1327	0.1120	0.3888	0.2321	0.3001	0.3476	0.3077
	SBFA($\mathcal{G}_1$)	0.1521	0.1283	0.1030	0.3857	0.2250	0.2903	0.3355	0.2921
	SBFA($\mathcal{G}_2$)	0.1432	0.1254	0.1001	0.3761	0.2201	0.2845	0.3310	0.2877
	SBFA($\mathcal{G}_3$)	0.1444	0.1254	0.0995	0.3770	0.2190	0.2830	0.3301	0.2876
	SBFA($\mathcal{G}_3^*$)	0.1481	0.1276	0.1007	0.3855	0.2243	0.2872	0.3360	0.2900
	JIVE	0.1525	0.1300	0.1101	0.8787	1.3423	1.2500	1.1653	0.6432
	SLIDE	0.1522	0.1319	0.1101	0.8770	1.5213	1.3245	1.1987	0.6510
	MOFA	0.1522	0.1320	0.1100	0.4423	1.4212	1.3013	1.1570	0.4623
II	s-GBFA($\mathcal{G}_0$)	0.1542	0.1331	0.1095	0.3900	0.2451	0.3045	0.3492	0.3143
	s-GBFA($\mathcal{G}_1$)	0.1420	0.1259	0.1000	0.3741	0.2120	0.2793	0.3265	0.2893
	s-GBFA($\mathcal{G}_2$)	0.1331	**0.1235**	**0.0995**	0.3630	0.2033	0.2656	0.3102	0.2765
	s-GBFA($\mathcal{G}_3$)	**0.1321**	0.1236	0.0996	**0.3621**	**0.2023**	**0.2577**	**0.3082**	**0.2733**
	s-GBFA($\mathcal{G}_3^*$)	0.1341	0.1244	0.0997	0.3722	0.2067	0.2704	0.3175	0.2830
	SBFA($\mathcal{G}_0$)	0.1557	0.1400	0.1138	0.4003	0.2577	0.3131	0.3567	0.3256
	SBFA($\mathcal{G}_1$)	0.1500	0.1302	0.1023	0.3881	0.2309	0.2888	0.3334	0.3155
	SBFA($\mathcal{G}_2$)	0.1441	0.1273	0.0997	0.3820	0.2259	0.2855	0.3301	0.3056
	SBFA($\mathcal{G}_3$)	0.1445	0.1277	0.0997	0.3789	0.2268	0.2861	0.3296	0.3021
	SBFA($\mathcal{G}_3^*$)	0.1475	0.1289	0.0997	0.3852	0.2331	0.2866	0.3321	0.3073
	JIVE	0.1667	0.1370	0.1010	0.8635	1.4019	1.2780	1.1324	0.6355
	SLIDE	0.1522	0.1319	0.1101	0.8770	1.5213	1.3245	1.1987	0.6510
	MOFA	0.1505	0.1344	0.1100	0.4325	1.4343	1.2643	1.1989	0.4722
III	s-GBFA($\mathcal{G}_0$)	0.1499	0.1367	0.1121	0.3903	0.2355	0.2985	0.3570	0.3147
	s-GBFA($\mathcal{G}_1$)	0.1423	0.1237	0.1001	0.3721	0.2175	0.2770	0.3244	0.2907
	s-GBFA($\mathcal{G}_2$)	0.1333	0.1235	**0.0995**	0.3643	**0.2103**	0.2657	0.3125	**0.2730**
	s-GBFA($\mathcal{G}_3$)	**0.1330**	**0.1231**	0.0996	**0.3634**	0.2104	**0.2611**	**0.3020**	0.2730
	s-GBFA($\mathcal{G}_3^*$)	0.1337	0.1242	0.0997	0.3700	0.2123	0.2669	0.3198	0.2801
	SBFA($\mathcal{G}_0$)	0.1530	0.1421	0.1210	0.4072	0.2444	0.3158	0.3737	0.3274
	SBFA($\mathcal{G}_1$)	0.1452	0.1320	0.1030	0.3890	0.2310	0.3032	0.3477	0.2973
	SBFA($\mathcal{G}_2$)	0.1403	0.1278	0.1003	0.3810	0.2256	0.2870	0.3401	0.2821
	SBFA($\mathcal{G}_3$)	0.1411	0.1270	0.0997	0.3796	0.2245	0.2853	0.3351	0.2831
	SBFA($\mathcal{G}_3^*$)	0.1441	0.1282	0.0997	0.3821	0.2272	0.2911	0.3433	0.2888
	JIVE	0.1701	0.1330	0.1157	0.8929	1.4489	1.3102	1.1529	0.6501
	SLIDE	0.1480	0.1347	0.1072	0.8765	1.3769	1.2454	1.1918	0.6482
	MOFA	0.1475	0.1324	0.1075	0.4506	1.3788	1.2345	1.1430	0.4745
IV	s-GBFA($\mathcal{G}_0$)	0.1451	0.1326	0.1085	0.3922	0.2349	0.2881	0.3455	0.3020
	s-GBFA($\mathcal{G}_1$)	0.1400	0.1265	0.0998	0.3755	0.2085	0.2730	0.3267	0.2910
	s-GBFA($\mathcal{G}_2$)	0.1340	0.1238	0.0996	0.3630	**0.2012**	0.2651	0.3082	**0.2703**
	s-GBFA($\mathcal{G}_3$)	**0.1330**	**0.1226**	**0.0995**	**0.3621**	0.2015	**0.2554**	**0.3032**	0.2710
	s-GBFA($\mathcal{G}_3^*$)	0.1344	0.1251	0.0996	0.3703	0.2087	0.2676	0.3170	0.2801
	SBFA($\mathcal{G}_0$)	0.1500	0.1373	0.1101	0.4131	0.2455	0.2979	0.3509	0.3200
	SBFA($\mathcal{G}_1$)	0.1431	0.1305	0.1030	0.4015	0.2244	0.2880	0.3299	0.3101
	SBFA($\mathcal{G}_2$)	0.1400	0.1277	0.1000	0.3930	0.2189	0.2821	0.3160	0.3027
	SBFA($\mathcal{G}_3$)	0.1370	0.1276	0.0996	0.3921	0.2172	0.2800	0.3151	0.3030
	SBFA($\mathcal{G}_3^*$)	0.1423	0.1284	0.0998	0.3957	0.2205	0.2872	0.3178	0.3067
	JIVE	0.1450	0.1320	0.1077	0.8666	1.3131	1.2567	1.1480	0.6332
	SLIDE	0.1452	0.1319	0.1056	0.8609	1.3402	1.2135	1.1423	0.6328
	MOFA	0.1447	0.1320	0.1063	0.4400	1.3799	1.2451	1.1151	0.4658

TABLE 2. The relative RE of the factor model for the s-GBFA and existing methods in the high dimensional setting ($p_1 = 90$,$p_2 = 60$,$p_3 = 30$, n=100). Four structures of W (Case I $\sim$ Case IV), three noise levels (large, medium and small), five graph structures ($\mathcal{G}_0 \sim \mathcal{G}_3^*$) and different data types are considered.

Method	RRE		MSE	
	Case I	Case IV	Case I	Case IV
s-GBFA	0.147	0.152	5.350	5.731
RABFA	0.153	0.167	5.877	6.790

TABLE 3. The RRE and MSE of the s-GBFA and RABFA in the high dimensional setting ($p_1 = 90, p_2 = 60, p_3 = 30, n = 200$) under graph $\mathcal{G}_2$. Two structures of W (Case I and Case IV) are considered. For s-GBFA, 5 outcomes are included.

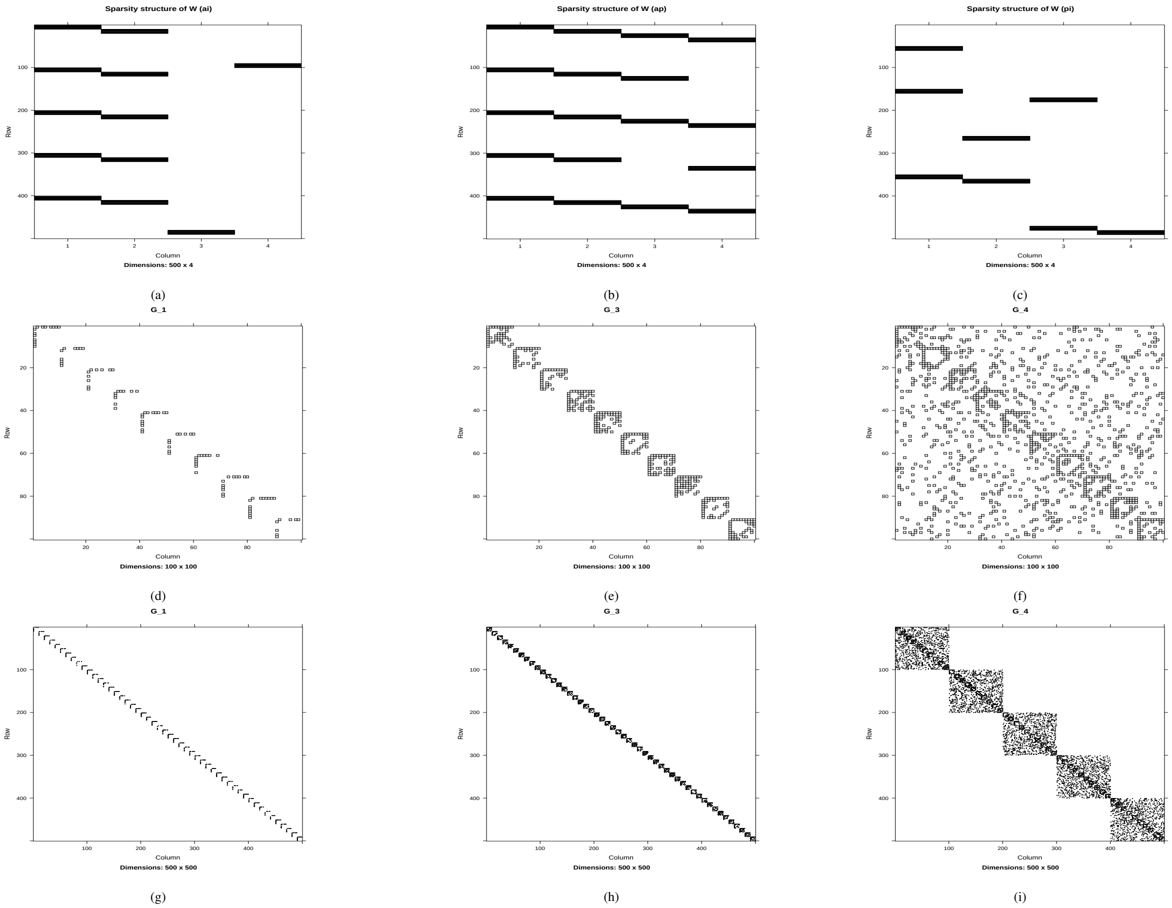

F I G U R E 2: Sparsity structures of the factor loading matrix (row 1), graph structure of $\mathcal{G}_1$, $\mathcal{G}_3$ and $\mathcal{G}_4$ for a single modality (row 2) and graph structure of $\mathcal{G}_1$, $\mathcal{G}_3$ and $\mathcal{G}_4$ for five modalities (row 3).
